# Characterization of dUTPase expression in mouse postnatal development and adult neurogenesis

**DOI:** 10.1038/s41598-024-63405-0

**Published:** 2024-06-07

**Authors:** Nikolett Nagy, Nóra Hádinger, Otília Tóth, Gergely Attila Rácz, Tímea Pintér, Zoltán Gál, Martin Urbán, Elen Gócza, László Hiripi, László Acsády, Beáta G. Vértessy

**Affiliations:** 1 Doctoral School of Biology, Institute of Biology, ELTE Eötvös Loránd University, Pázmány Péter sétány 1/C, 1117 Budapest, Hungary; 2 Institute of Molecular Life Sciences, Research Centre for Natural Sciences, HUN-REN, Magyar tudósok körútja 2, 1117 Budapest, Hungary; 3 Laboratory of Thalamus Research, Institute of Experimental Medicine, HUN-REN, Szigony utca 43, 1083 Budapest, Hungary; 4 Department of Applied Biotechnology and Food Sciences, Faculty of Chemical Technology and Biotechnology, BME Budapest University of Technology and Economics, Műegyetem rkp. 3, 1111 Budapest, Hungary; 5 Department of Animal Biotechnology, Institute of Genetics and Biotechnology, Hungarian University of Agriculture and Life Sciences, Szent-Györgyi Albert utca 4, 2100 Gödöllő, Hungary; 6 Laboratory Animal Science Coordination Center, Semmelweis University, Nagyvárad tér 4, 1089 Budapest, Hungary

**Keywords:** Cell proliferation, Differentiation, Neurogenesis, Organogenesis, Mouse, Reverse transcription polymerase chain reaction

## Abstract

The enzyme dUTPase has an essential role in maintaining genomic integrity. In mouse, nuclear and mitochondrial isoforms of the enzyme have been described. Here we present the isoform-specific mRNA expression levels in different murine organs during development using RT-qPCR. In this study, we analyzed organs of 14.5-day embryos and of postnatal 2-, 4-, 10-week- and 13-month-old mice. We demonstrate organ-, sex- and developmental stage-specific differences in the mRNA expression levels of both isoforms. We found high mRNA expression level of the nuclear isoform in the embryo brain, and the expression level remained relatively high in the adult brain as well. This was surprising, since dUTPase is known to play an important role in proliferating cells, and mass production of neural cells is completed by adulthood. Thus, we investigated the pattern of the dUTPase protein expression specifically in the adult brain with immunostaining and found that dUTPase is present in the germinative zones, the subventricular and the subgranular zones, where neurogenesis occurs and in the rostral migratory stream where neuroblasts migrate to the olfactory bulb. These novel findings suggest that dUTPase may have a role in cell differentiation and indicate that accurate dTTP biosynthesis can be vital, especially in neurogenesis.

## Introduction

The deoxyuridine 5′-triphosphate nucleotidohydrolase, dUTPase, is important during DNA replication and repair, ensuring the fidelity of DNA synthesis and the maintenance of genome stability. By its catalytic activity dUTPase hydrolyses dUTP into dUMP and inorganic pyrophosphate preventing the incorporation of uracil into the DNA. Besides providing dUMP, the substrate of thymidylate synthase, the dUTP/dTTP ratio is controlled in the cells by this enzyme^1^. As DNA polymerases cannot distinguish between uracil and thymine, if dUTP is available in the nucleotide pool, it will be incorporated into the genome. Whereas this process in not mutagenic, uracil can appear in the DNA through cytosine deamination as well, which is a mutagenic reaction. Uracil bases are excised via the base excision repair (BER) mechanism initiated by uracil-DNA-glycosylases irrespectively of their source. If the dUTP/dTTP ratio is high, the recurring incorporation of dUTP leads to a futile repair cycle and can induce single and double stranded breaks in the DNA, which may cause cell death.

Two isoforms of the human dUTPase enzyme have been described, a nuclear and a mitochondrial isoform. These isoforms are encoded by the *DUT* gene and are generated by alternative splicing coupled with alternative promoter usage^2,3^. In mice as well, these two isoforms exist. The nuclear isoform is more abundant and localises to the nucleus, while the mitochondrial isoform contains a mitochondrial targeting signal and is transported to the mitochondria. It was shown that knock-out of the *Dut* gene leads to early embryonic lethality in mouse, since embryos with *Dut*−/− genotype die after the blastocyst stage^4^. In order to describe the mRNA expression levels of the nuclear (nDut) and the mitochondrial (mDut) isoforms in adult mouse organs, we developed an RT-qPCR method^5^.

Mouse development is an extensively studied process that serves as a useful model for understanding mammalian development^6,7^. The enzymatic activity of dUTPase during development has been described in rabbit brain and liver^8,9^, while during mouse development it was investigated using in situ hybridization (ISH) and immunohistochemistry^10^. Nevertheless, only high-throughput transcriptome data are available to describe the expression of dUTPase in various mouse organs and these results are often diverse and do not distinguish the sexes^11,12^. Moreover, there is a lack of knowledge about the quantitative expression of the different isoforms of dUTPase during development.

Expression of nDut in the adult brain has not been characterized in detail. A large, comprehensive database containing data generated by ISH to determine the genome-wide gene expression profile of the adult mouse brain (Allen Mouse Brain Atlas)^13^ shows no detectable level of the *Dut* gene in adults. In the St. Jude Brain Gene Expression Map (BGEM), dUTPase is detected in several regions of the brain during development in mouse using ISH^14^. In addition, it has been shown in perinatal rat neurons that the enzymatic activity of dUTPase is high during neuronal development^15^. In rabbits, enzymatic activity of dUTPase was analysed in different brain regions during development. It was shown that enzymatic activity of dUTPase is not restricted to the developing brain, however, the activity is higher in newborn and 1-week-old whole brain than in the adult brain^9^. The expression of dUTPase in different cell types of the brain was not characterised further in the literature.

Here we demonstrate the isoform-specific mRNA expression of dUTPase in various organs of mice during development using RT-qPCR. We investigated organs derived from 14.5-day embryos and from 2-week-old, 4-week-old, 10-week-old and 13-month-old mice to represent the milestones during development using FVB/N mouse strain. The elevated expression of nDut observed in the adult mouse brain was unexpected, as dUTPase is known to play an important role in proliferating cells and mass production of neural cells is completed by adulthood. Furthermore, it was shown previously that appropriate dTTP biosynthesis is essential in neuronal development, since in human and in zebrafish erroneous thymidylate kinase (DTYMK) led to severe postnatal neurodegenerative disease^16,17^. As dUTPase has a central role in de novo dTTP biosynthesis, we aimed to characterize the protein expression of dUTPase in the 3-month-old adult mouse brain with immunostaining.

## Results and discussion

### Isoform-specific dUTPase expression during development

We investigated brain, gonad and liver samples derived from 14.5-day embryos. Furthermore, heart, kidney, lung, spleen and thymus samples were also investigated in 2-week-old, 4-week-old, 10-week-old and 13-month-old mice. With the analysis of these organs, we could cover the three germ layers: brain (ectoderm); heart, kidney, ovary and testis (mesoderm); thymus, lung and liver (endoderm). The investigated developmental stages represent the milestones during mouse development. At 14.5 days post-fertilization (E14.5), embryos are in the mid-gestation stage undergoing advanced developmental processes, while after birth at 2 weeks of age mice are considered neonates. Puberty begins after 4 weeks when mice are separated from their mother; and after 10 weeks, mice reach sexual maturity; while at 13 months of age, mice are reckoned relatively old.

In our previous study, mRNA expression of the mitochondrial and the nuclear isoforms of dUTPase were measured using RT-qPCR^5^. We found that the expression of the nuclear isoform varies greatly, while the mitochondrial isoform has considerably more stable expression among various organs. In adult mice the nuclear isoform is expressed at a high level in thymus, in spleen and in reproductive organs^5^. These results are in good accordance with the well-established role of dUTPase in dividing cells, hence in these organs extensive mitosis occurs. Cell proliferation is due to lymphocyte production in the thymus and in the spleen, while it is due to spermatogenesis in the testis, and folliculogenesis in the ovary.

The relative expression values of both isoforms were determined in the organs of 10-week-old mice without using reference genes in our previous study. Application of this approach is adequate as the variance of the Cq values measured using three biological replicate samples and three technical replicate measurements for each sample was considerably lower (below 0.2 cycles) in case of each isoform. However, for the reliable determination of expression levels during development, the use of reference genes was important. For this purpose, we chose to determine the gene expression levels of *Gapdh* and *Ppia* as well, since both genes were shown to be adequate as reference in the context of mouse organ development^18–27^. It is important to note that these reference genes were not used to compare expression data between organs. Alternatively, the relative normalized expression values, measured during development of each organ, were corrected with the relative expression values of the 10-week-old organs^5^. This approach allowed us to compare the relative normalized expression data between the organs for each isoform without introducing bias to the results, as the expression of these reference genes may vary among organs. The variance of the expression within biological replicate samples was not equal, since during development gene expression can vary greatly. Thus, for the statistical analysis we performed non-parametric Kruskal–Wallis tests followed by Conover-Iman pairwise comparisons with Bonferroni correction using an overall 5% significance level. Based on these criteria all changes in the expression values described below are considered significant unless otherwise noted. The Bonferroni corrected significance level in case of the brain, gonad and liver was 0.0018 as E14.5 samples were also analysed, while in case of the heart, kidney, lung, spleen and thymus it was 0.0024.

**Figure 1:**
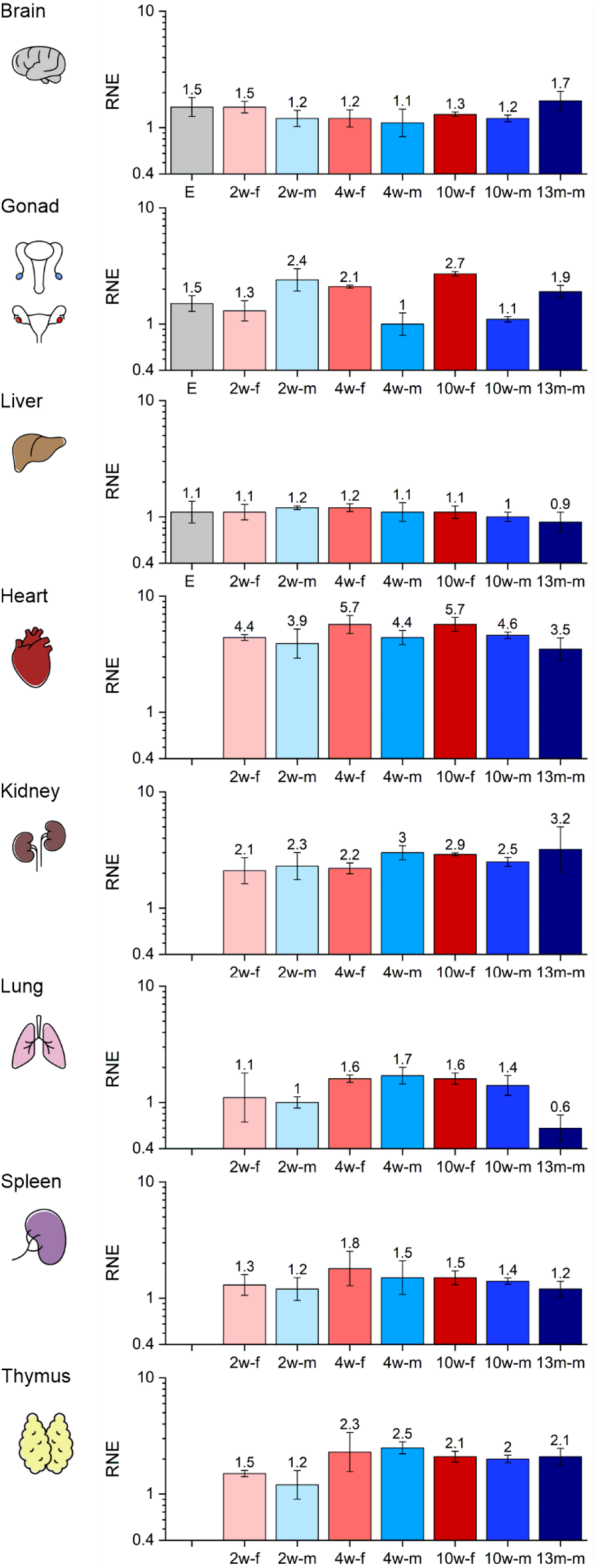
The relative normalized expression (RNE) of the mitochondrial isoform of dUTPase (mDut) in different organs during development. Organs are illustrated schematically. In every case the same logarithmic scale is used from 0.4 to 10. The biological groups are abbreviated and colored as E: 14.5-day embryo (light grey), 2w-f: 2-week-old female (pale pink), 2w-m: 2-week-old male (pale blue), 4w-f: 4-week-old female (pale red), 4w-m: 4-week-old male (light blue), 10w-f: 10-week-old female (red), 10w-m: 10-week-old male (blue), 13m-m: 13-month-old male (dark blue). The error bars represent standard deviation of the biological replicate samples (for the number of the individual replicates see Supplementary Table S1). Expression data regarding the 10-week-old stage have been described previously^5^. Individual graphs were created with OriginPro 2018 (OriginLab Corp.) and the figure was assembled using CorelDRAW Graphics Suite 2021 (Corel Corporation).

Regarding the mRNA expression of mDut (Fig. 1), it was the highest in the heart and kidney, which organs are known to be rich in mitochondria^28^. We found no significant changes in the expression during development and between sexes in case of the brain, liver, kidney and spleen. However, a slight, although not significant increase in the expression in the 13-month-old brain can be observed. DNA damage is believed to play an important role in the aging process. It was shown that the activity of DNA glycosylases, particularly uracil-DNA-glycosylases was significantly decreased in the mitochondrial BER process in different brain regions during aging in mice^29^ and in rats^30^. It was also shown in a proteomic analysis in mouse brain mitochondria, that changes in mitochondrial protein expression may act as a compensatory mechanism allowing for preservation of function during aging^31^. Accordingly, we suggest that mDut expression may be elevated in the aging brain to maintain the integrity of the mitochondrial genome via the preventive sanitizing role of dUTPase.

In the reproductive organs great differences can be described during development and between sexes as well (Fig. 1). We observed significant differences between the sexes at the 2-, 4- and 10-week-old stages. At the 2-week-old stage the expression was higher in the testis than in the ovary, however, at the 4- and 10-week-old stages the expression in the ovary was higher than in the testis. In male mice, the expression was higher at the 2-week-old stage than in the E14.5 stage, however, a decrease was observed by the 4-week-old stage. Between the 4- and 10-week-old stages the expression remained unchanged, but a slight increase could be observed in the 13-month-old testis. Mitochondria undergo regulated fusion, fission and ubiquitination during cell division and differentiation to guarantee cellular energy demands and normal mitochondrial inheritance^32,33^. During spermatogenesis, germ cells differentiate to form haploid round spermatids which mature to functional elongated spermatozoa and lose a large fraction of cellular proteins via ubiquitination. In rat testis the amount of ubiquitin conjugates, the rate of ubiquitination and the polyubiquitin mRNA level were shown to be the lowest in the 10-day-old samples and were greatly increased with a peak in the 30-day-old samples^34^. In addition to the high level of ubiquitination, expression of proteins responsible for mitochondrial fusion (mitofusins 1 and 2, Mfn1, Mfn2) and fission (dynamin-related protein 1, Drp1) were found to be markedly elevated in testis of pubertal (4-week-old) and adult (7-week-old) rats, suggesting that at a given stage of spermiogenesis fusion and fission should be greatly increased for achieving complete homogenization^35^. These results are in good accordance with our observations regarding the decrease in mDut expression in the testis at puberty, when both ubiquitination and fusion and fission events occur. However, other processes may contribute to the observed slight increase in the 13-month-old testis. As aging is associated with the impairment of mitochondrial BER and mitochondrial DNA instability^36^, we assume that the increase in mDut expression may compensate the decrease in uracil-DNA-glycosylase activity. In female mice, there was no difference between the E14.5 gonad and the 2-week-old ovary samples. Expression in the ovary increased by the 4-week-old stage, however, did not change between the 4- and 10-week-old stages. It is well established that mitochondria play a central role in oocyte maturation, fertilization and embryonic development^37,38^. In humans, an adult primordial follicle oocyte contains approximately 6000 mitochondria^39^. Moreover, during later stages of folliculogenesis, the number of mitochondria increases greatly. In contrast, a typical mammalian sperm midpiece contains approximately 50–75 mitochondria^40^. These data suggest that elevation in the expression of mDut is related to a high number of mitochondria of the oocytes in the ovaries of mature mice.

In case of the heart, we observed no differences in the expression levels between sexes and during development, however, expression of mDut in the 4- and 10-week-old female groups was higher than in the 13-month-old male mice. The heart is a powerful organ that drives the circulation and therefore requires a large amount of energy. Function of the heart is strictly dependent on ATP synthesis; hence mitochondria provide the energy needed and make up more than 35% of the total cardiomyocyte volume in mouse^41,42^. The relative amount of mitochondrial DNA is the highest in the heart throughout the entire lifespan in mice^43^. Importantly, mitochondrial function and ultrastructure of mouse heart are maintained during aging^44^. Accordingly, we observed the highest expression of mDut in the heart during postnatal development. Moreover, in every developmental stage investigated the expression of mDut was comparable to the nDut according to the amplification curves (Supplementary Fig. S1).

Regarding the lung, the only significant difference in the expression was a decrease in the 13-month-old male mice compared to the 4-week-old male and female group. Lung function and structure are altered most between 3 and 12 months of age. Tissue elastance and tissue resistance are significantly decreased with age. Interestingly, these changes are paralleled with a significant decrease in alveolar volume and alveolar epithelial type II (ATII) cells^45^. Decrease in the cell number may provide some explanation for the observed decrease in both mitochondrial and nuclear isoforms of dUTPase.

In case of the thymus, the expression of mDut in the 2-week-old female mice was lower than in the 4-week-old males, while the expression in the 2-week-old males was lower than in the 4-week-old groups, in the 10-week-old females and in the 13-month-old males. The thymic cell number increases and reaches its maximum at 4 weeks of age in mouse and decreases during aging^46^. In the early postnatal development, the thymic vasculature matures into an adult form, when the cortical thymic epithelial cells undergo a transition to medullary thymic epithelial cells^47^. These age-related changes in thymus maturation may correlate to the observed increase in the mDut expression from 2-week-old to 4-week-old mice.

Overall, the observed changes in the mDut expression between tissues and during development were lower than threefold in all cases. Our results are in good accordance with the data found in the Mouse MitoCarta3.0 reference inventory of mitochondrial proteins, in which the dUTPase protein expression showed nearly constant values among the tissues investigated^48^.

**Figure 2:**
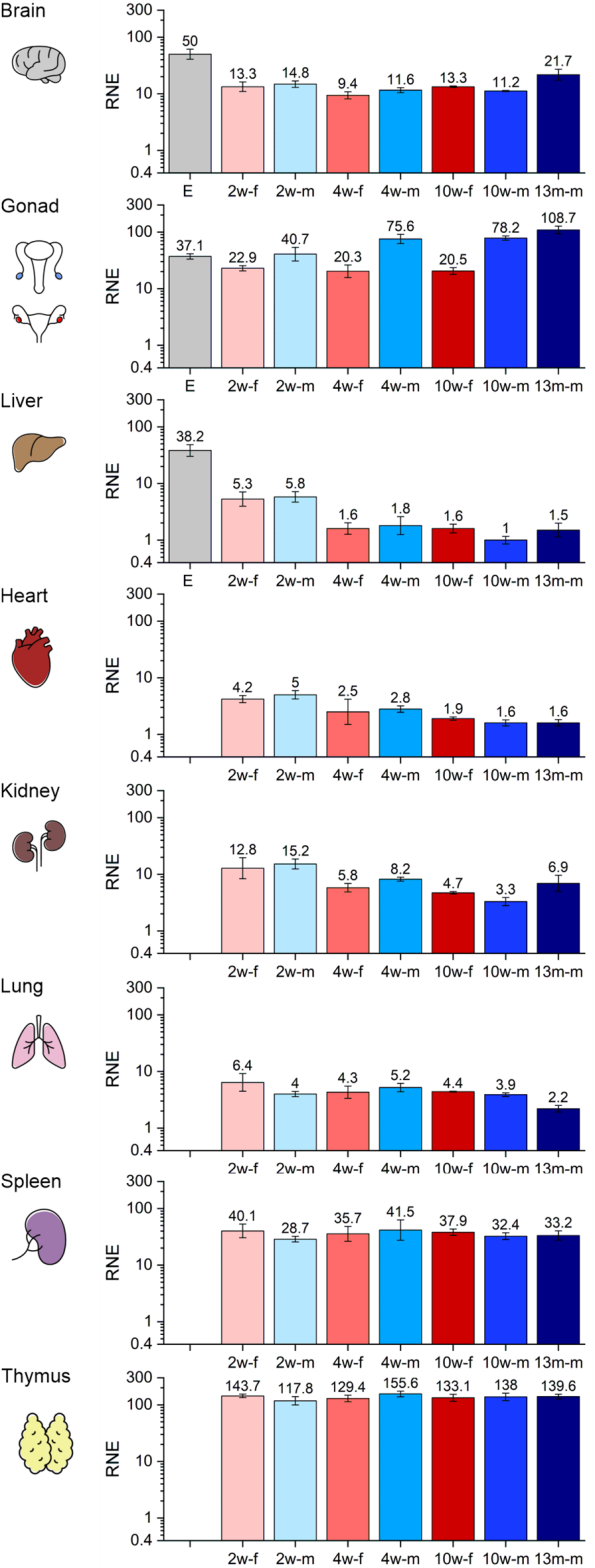
The relative normalized expression (RNE) of the nuclear isoform of dUTPase (nDut) in different organs during development. Organs are illustrated schematically. In every case the same logarithmic scale is used from 0.4 to 300. The biological groups are abbreviated and colored as E: 14.5-day embryo (light grey), 2w-f: 2-week-old female (pale pink), 2w-m: 2-week-old male (pale blue), 4w-f: 4-week-old female (pale red), 4w-m: 4-week-old male (light blue), 10w-f: 10-week-old female (red), 10w-m: 10-week-old male (blue), 13m-m: 13-month-old male (dark blue). The error bars represent standard deviation of the biological replicate samples (for the number of the individual replicates see Supplementary Table S1). Expression data regarding the 10-week-old stage have been described previously^5^. Individual graphs were created with OriginPro 2018 (OriginLab Corp.) and the figure was assembled using CorelDRAW Graphics Suite 2021 (Corel Corporation).

The mRNA of the nuclear isoform of dUTPase is more abundant than the mitochondrial isoform, as indicated by the lower Cq values measured for the nuclear isoform. In addition, changes in the expression during development and among the organs are markedly higher (Fig. 2).

The mRNA expression of nDut was the highest in the thymus and spleen, and these results are in good accordance with RNA-Seq transcriptome data^49–51^ and with dUTPase activity measured in rat thymus and spleen^52^. Interestingly the expression did not change during development in both organs, thus, high level of dUTPase expression is uniformly ensured during the entire lifespan of the animal (Fig. 2). It has been described that in human thymus, the expression of dUTPase occurs in a cell cycle-dependent manner in mature T cells, however, in immature thymocytes the expression is constitutively high and is independent of proliferation^53^. The constantly high expression level of nDut observed during development may be explained by the fact that maturation of thymocytes occurs in the thymus and immature thymocytes reside there^54^. Similarly, in the spleen early maturation and differentiation process of B cells occurs^55^.

Regarding the expression of nDut in the liver, remarkable changes can be observed during development (Fig. 2). The expression was significantly higher in the E14.5 embryos and in the 2-week-old mice than at the other developmental stages. However, by the 4-week-old stage the expression decreased and remained on this level throughout the development, which is the lowest expression level among the organs investigated. Highlighting the difference, in the embryo liver the expression of nDut was more than 20-fold higher than in the 4- and 10-week-old liver samples. During embryonic development hepatogenesis is regulated by a network of signaling pathways, growth factors and transcription factors^56^. The two major epithelial cell types of liver, hepatocytes and cholangiocytes are differentiated from liver stem/progenitor cells (LSPCs) during embryonic liver development, and E14.5 is a transition point when these cells develop from the bipotential hepatoblasts. Many cell cycle- and mitosis-related genes have elevated expression in E14.5 hepatoblasts, suggesting that these cells may be induced to initiate differentiation via cell division^57,58^. Moreover, it has been shown in regenerating rat liver that dUTPase activity is highly increased^59^. Our results, regarding the highly elevated expression level of nDut in the embryonic liver, suggest that dUTPase may have an important role in cell differentiation.

In the reproductive organs the mRNA expression of nDut was also relatively high. During development, expression in the ovary did not change, however, in the E14.5 gonad samples the expression was significantly higher than at the other investigated developmental stages. On the other hand, the expression in the E14.5 gonad samples was lower than in case of the adult testis. At the 2-week-old stage no differences between the sexes can be observed, but at the start of puberty at the 4-week-old stage, differences arised, i.e., in the testis the expression of nDut was higher than in the ovary. Compared to the 4-week-old ovary, the expression in the 2-week-old testis was twofold higher, while in the 4-week-old testis the expression was more than threefold higher. These differences remained in the adult mice as the expression did not change significantly between the 4- and the 10-week-old stages (Fig. 2). Correspondingly, according to transcriptome data the expression of dUTPase in the testis is high^49–51,60,61^. Gonad differentiation is tightly regulated by the expression of specific genes and hormones^62^. Although differentiation starts before E14.5, we did not distinguish the male and female gonad samples. The variance of the expression data indicates that there is no marked difference in the expression of the dUTPase isoforms between the sexes at this stage. During oogenesis, oocytes increase in size and in volume and synthesize and accumulate transcripts and proteins, which are essential for oocyte competence and are indispensable for its development into a viable embryo. Murine oocytes have been estimated to contain approximately 200 times more RNA than a typical somatic cell. During folliculogenesis, the oocyte matures in interaction with its associated somatic cells. Communication between the oocyte and the surrounding granulosa cells is essential for both oocyte development and granulosa cell differentiation^63,64^. Elevated and stable expression level of nDut in the ovary from the embryonic until the adult stage may be caused by the transcript accumulation in the oocytes and the maturation process via folliculogenesis, as no mitotically active female germline progenitors exist in postnatal mouse ovaries^65,66^. However, in the testis spermatogonial stem cells (SSCs) are known to be mitotically active^67^. During spermatogenesis SSCs maintain stem cell pool by self-renewing divisions, in addition, sustain continuous sperm production via differentiating divisions^68^. During spermatogenesis dUTPase activity was found to be maximal at the pachytene stages^69^. As during spermatogenesis continuous renewal of spermatocytes occurs, this may be the reason why the expression of nDut in the adult testis is threefold higher than in the adult ovary. In the 13-month-old testis the expression of nDut showed a slightly, but not significantly elevated level than in the 10-week-old testis, however, the expression was higher than in the 10-week-old ovary. In the testis maintaining the germ line genomic stability is of utmost importance, accordingly BER gene transcripts are reported to be highly expressed^70,71^. It is also stated that BER activity decreases during aging in mouse germ cells^72^. Similarly to mDut, an increase in the nDut expression during aging may contribute to maintain genomic integrity in male germ cells by compensating the decline in uracil-DNA-glycosylase expression.

In case of the heart, the expression was higher in both sexes at the 2-week-old stage relative to the 10-week-old and 13-month-old male samples. It was shown that the proliferation rate of cardiac fibroblast cells was remarkably high in the neonatal period, then rapidly decreased to the 4-week-old stage, and declined further until adulthood to a stable and low level remaining similar in 1-year-old animals^73,74^. These postnatal changes in the fibroblast proliferation are strongly associated with the observed alteration of nDut expression in the mouse heart, further supporting the role of dUTPase in cell proliferation.

Regarding the kidney, there were no changes between the sexes at any developmental stage investigated. However, the expression in the 2-week-old groups was higher than in the 4-week-old females and 10-week-old groups. The expression in the 10-week-old males was lower than in the 4-week-old males and the expression of the 13-month-old males was lower than in the 2-week-old males, and a slight, although not significant increase can be observed from the 10-week-old stage to the 13-month-old stage. The cellular composition of each tissue tends to vary with age. Single cell transcriptome data are useful to assess overall changes in tissues with age. According to the Mouse Cell Atlas 3.0 RNA-sequencing database, *Dut* expression is elevated in the ascending loop of Henle, in the distal convoluted tubule, and in connecting tubule cells^75^. Histologic studies showed that the size of renal corpuscles, glomerular tufts and nuclei of glomerular and tubular cells were increased during aging in mouse kidney^76^. These findings of the expanding medullary volume correlate with our observations regarding the elevated expression of nDut in old mouse kidney.

In the lung, there were no differences neither between the sexes nor between the developmental stages, only the 13-month-old male mice showed lower expression than the 2-week-old female mice. It was shown that in early postnatal rat lung development intense cell proliferation and differentiation happen, when alveolar epithelial type II (ATII) cell number starts to decrease and alveolar epithelial type I (ATI) cell number increases^77^. ATII cells serve as progenitor cells for ATI cells^78^. In mouse lung, ATII cell number significantly decreases between 3 and 12 months of age^45^. The consistent decline in the cell number reinforces our results, regarding the observed decrease in nDut expression between 2-week-old female and 13-month-old male lung.

In case of the brain, there were no differences between the sexes at any developmental stage investigated. The expression was the highest in the E14.5 samples, which was higher than in the 2-week-old female and in the 4- and 10-week-old groups (Fig. 2). The expression measured in the 4-week-old female brain was lower than in the 2-week-old male and in the 10-week-old female brain samples. Interestingly, in the 13-month-old male brain the expression was slightly higher than in the 4-week-old groups and in the 10-week-old males. Accordingly, transcriptome data indicate high dUTPase expression in the whole brain of adult mice^49,51,60,61,79,80^. Genome-wide sequencing data reveal dUTPase expression in dentate gyrus, olfactory bulb, striatum and subventricular zone in the adult mouse brain^81^. Regarding the slight increase in the expression of nDut in the 13-month-old brain, aging related cellular processes may be responsible. Various studies have reported an age-related decline in DNA repair processes, especially in the BER pathway in mouse brain^29,82^. Increase in the nDut expression might be important to sustain integrity of the genome, even when uracil-DNA-glycosylase activity is compromised.

### Protein expression of dUTPase in the adult mouse brain

**Figure 3:**
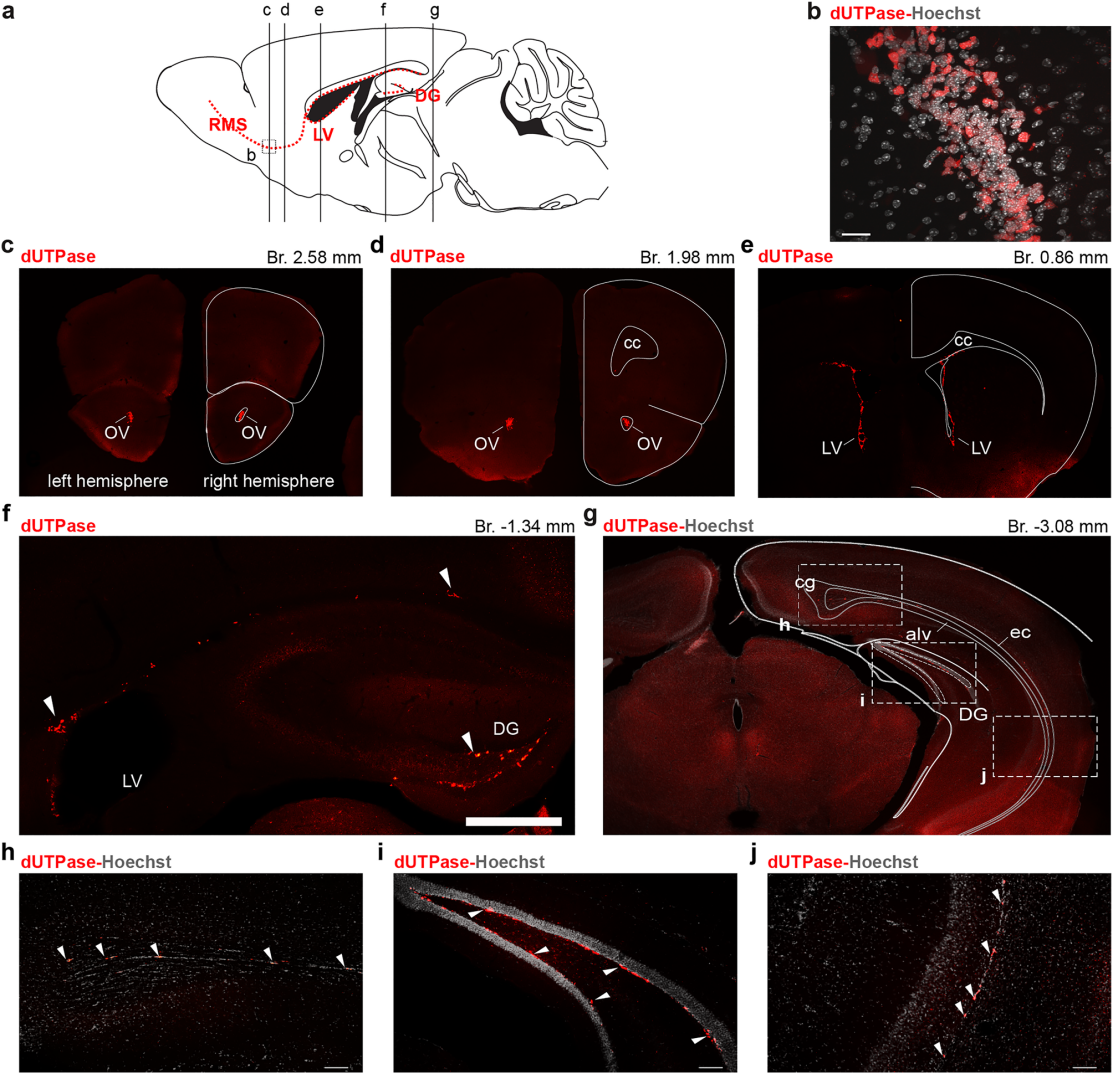
Expression of dUTPase throughout the neurogenic niches and in the rostral migratory stream (RMS) in 3-month-old adult mouse brain. (**a**) Schematic horizontal view of the mouse brain at interaural 0.48 mm. Neurogenic niches, lateral ventricle (LV) and dentate gyrus (DG) and the RMS are labelled by red dashed lines. Antero-posterior levels of coronal samples on (**c–g**) are indicated by vertical lines. (**b**) High resolution confocal image of the dUTPase staining (red) along the RMS. Cell nuclei are stained with Hoechst (gray). Scale bar: 20 μm. (**c**) Low resolution fluorescent image of a coronal brain section + 2.58 mm from the Bregma immunostained for dUTPase (red). On the right hemisphere, outlines of the brain and position of the olfactory ventricle (OV) are indicated. (**d,e**) Same as (**c**) at + 1.98 mm and + 0.86 mm from the Bregma respectively. *cc* corpus callosum. (**f**) dUTPase immunostaining (red) in the wall of the LV (subventricular zone, SVZ) and in the DG (subgranular zone, SGZ) at coronal level − 1.34 mm from the Bregma. Arrowheads point to dUTPase positive cell groups. Scale bar: 500 μm. (**g**) Low resolution fluorescent image at − 3.08 mm from the Bregma. dUTPase (red), Hoechst staining (gray). Regions of interest (ROIs) for (**h–j**) are indicated. *cg* cingulum, *alv* alveus, *ec* external capsule. (**h–j**) Higher resolution images for regions indicated on (**g**). Arrowheads point to dUTPase positive cell groups. Scale bar: 20 μm. Individual images were captured with a Nikon AR1 confocal microscope, and the figure was assembled using Adobe Illustrator (Adobe).

Our RT-qPCR data suggested that both mitochondrial and nuclear isoforms of the dUTPase enzyme are present in the adult mouse brain. Therefore, we aimed to detect dUTPase at the protein level in 3-month-old mouse brain and to determine the localization of the enzyme in different brain regions, cell types and at subcellular levels. Thus, we carried out immunostaining of brain sections (n = 4 mice) and examined the results using confocal microscopy. In the adult mammalian brain extensive neurogenesis is described in the subventricular zone (SVZ) located along the walls of the lateral ventricles (LV) and in the subgranular zone (SGZ) of the hippocampal dentate gyrus^83^. In the SVZ neural stem cells (NSCs) generate neuroblasts, which migrate through the rostral migratory stream (RMS) into the olfactory bulb (OB) where they differentiate into functional interneurons (Fig. 3a)^84,85^. We found that intensive dUTPase labelling was mainly restricted to these adult germinative zones (Fig. 3c–j) and to the RMS along which neuroblasts from the SVZ migrate to reach the OB (Fig. 3b–d). According to a recent study, thousands of new OB neurons are generated every day in the young adult rodent brain^86^. Although the observed dUTPase protein expression is limited to the germinative zones, as several thousands of cells proliferate and migrate expressing the protein, these observations may account for the measured high mRNA expression level of dUTPase in the brain. Interestingly, labelled cells could be also found in regions, where during the development the cavity of the LV disappears between the alveus and the external capsule (Fig. 3e–g,i).

The expression of dUTPase protein in the adult neural germinative zones correlates with the well-known role of dUTPase in the proliferating cells, i.e., preserving the integrity of the genome by sanitizing the nucleotide pool from dUTP^87^. In addition, it was shown that the enzymatic activity of dUTPase is not restricted to the developing brain in rabbit, however, the activity is higher in the newborn and 1-week-old whole brain than in the adult brain. Furthermore, the enzymatic activity was the highest in the developing cerebellum in the 1-week-old rabbit brain. In the adult rabbit brain similar activity was measured in the forebrain, in the cerebellum and in the brainstem compared to each other, nevertheless, the activity was higher in the midbrain and diencephalon^9^.

**Figure 4:**
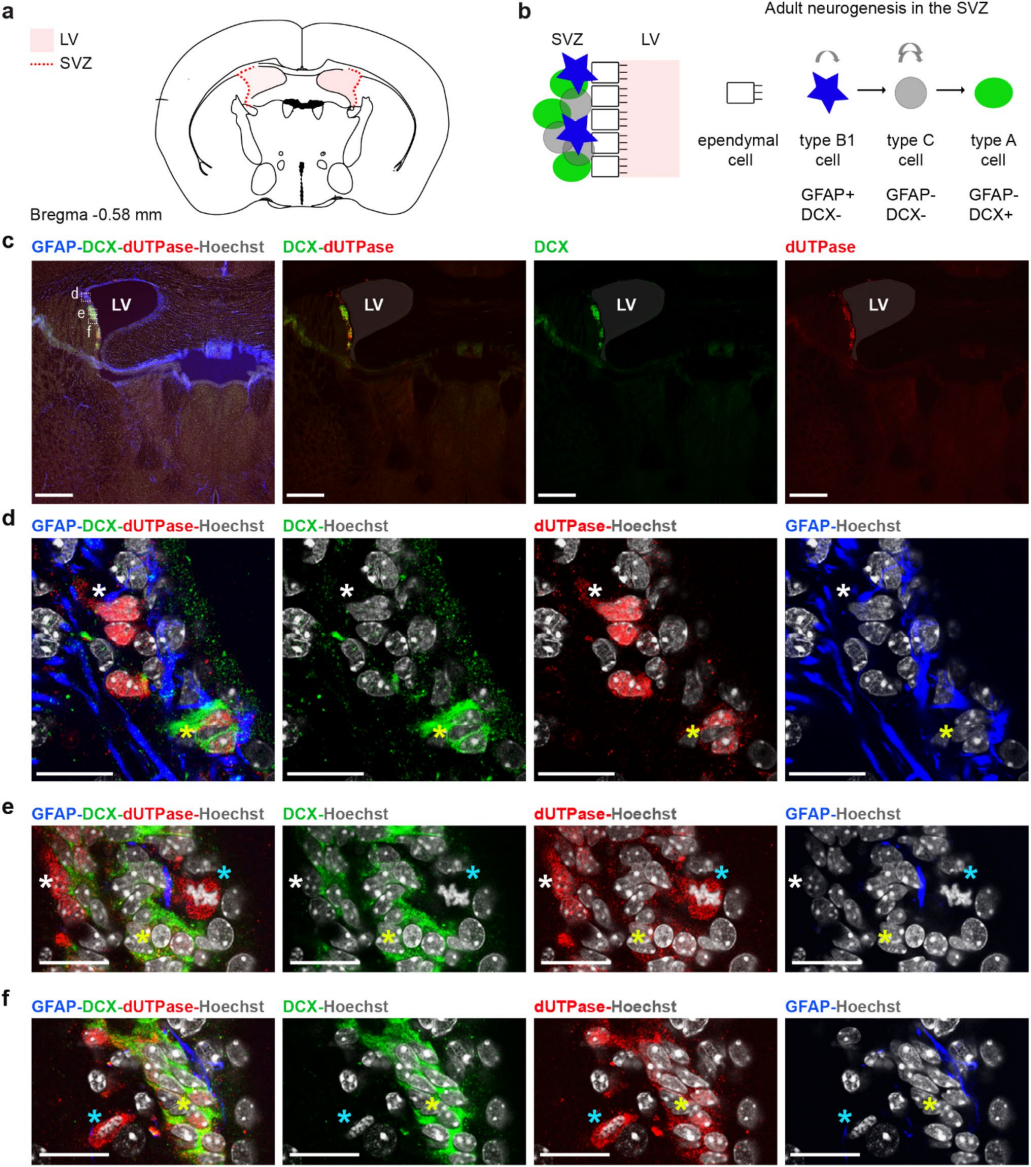
Expression of dUTPase in the subventricular zone (SVZ) in 3-month-old adult mouse brain. (**a**) Position of the SVZ at the coronal level matching the regions of interest (ROIs) of (**c–f**). *LV* lateral ventricle (**b**) Schematic image illustrating the cellular composition of the SVZ (left panel) and the lineage (type B1 cell: relatively quiescent neural stem cell, type C cell: transit amplifying cell, type A cell: neuroblast) and antigenic properties (GFAP glial fibrillary acidic protein, DCX doublecortin) of the SVZ progenitor cells (right panel). (**c**) Low resolution confocal image of a coronal brain section containing the SVZ, immunostained for GFAP (blue), DCX (green), dUTPase (red) and Hoechst (grey). Left panel: merged image with ROIs of (**d–f**) panels (dashed lines). Middle and right panels: dUTPase immunostaining colocalizes with the DCX immunostaining. Both stainings are restricted to the SVZ. Scale bar: 200 μm. (**d–f**) High resolution confocal images of the SVZ. Immunostainings for GFAP (blue), DCX (green) and dUTPase (red). Nuclei of the cells are labelled by Hoechst staining (grey). Left panel: merged image. Middle and right panels: single channels merged with Hoechst label. Scale bar: 20 μm. (**d**) Nuclear dUTPase staining could be observed in both DCX+/GFAP− cells (neuroblasts, yellow asterisk) and DCX−/GFAP− cells (putative transit amplifying cells, white asterisk). (**e,f**) dUTPase staining could be detected in DCX−GFAP− mitotic cells (putative transit amplifying cells) where Hoechst staining revealed condensed chromatin structure (blue asterisk). Yellow asterisks show DCX+/GFAP− cells (neuroblasts) with nuclear and/or cytoplasmal dUTPase labelling. White asterisks label DCX−/GFAP− cells (putative transit amplifying cells) with dUTPase staining in the nucleus. Individual images were captured with a Nikon AR1 confocal microscope, and the figure was assembled using Adobe Illustrator (Adobe).

**Figure 5:**
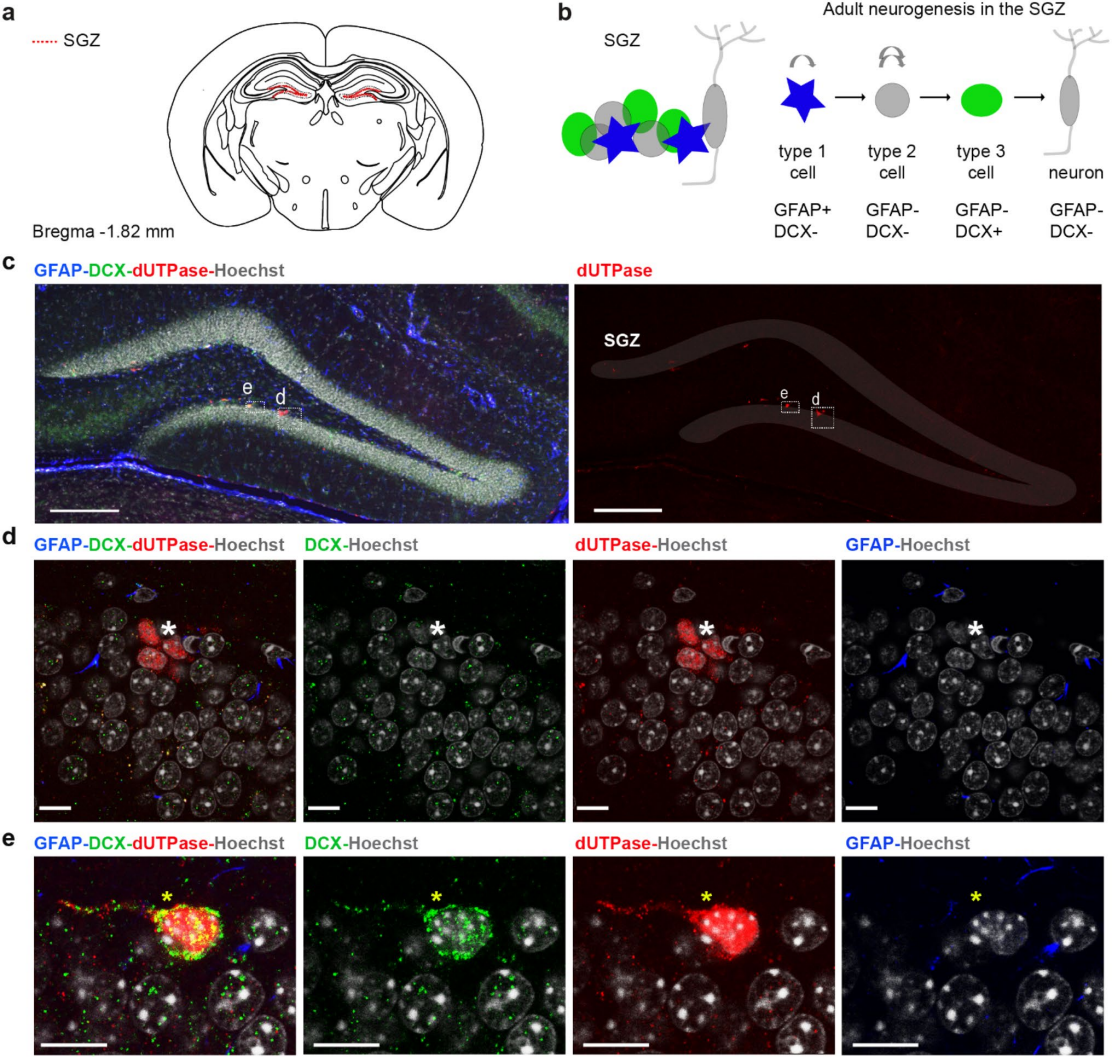
Expression of dUTPase in the subgranular zone (SGZ) of the dentate gyrus in 3-month-old adult mouse brain. (**a**) Schematic figure of the mouse brain at the coronal level matching the sampling level of (**c–e**). The position of the SGZ is indicated by dashed red lines. (**b**) Schematic figure illustrating the cellular composition of the SGZ (left panel) and the lineage (type 1 cell: relatively quiescent neural stem cell, type 2 cell: transit amplifying cell, type 3 cell: neuroblast) and antigenic properties (GFAP glial fibrillary acidic protein, DCX doublecortin) of the SGZ progenitor cells (right panel). (**c**) Low resolution confocal image of a coronal section of the dentate gyrus immunostained for GFAP (blue), DCX (green), dUTPase (red), and Hoechst (grey). Left panel: merged image with regions of interest (ROIs) of (**d,e**) panels (dashed white lines). Right panel: dUTPase immunostaining is restricted to the SGZ. ROIs of (**d,e**) panels are delineated by dashed lines. Granular layer is shaded in grey. Scale bar: 200 μm. (**d,e**) High resolution confocal image of the SGZ. Immunostainings for GFAP (blue), DCX (green) and dUTPase (red). Nuclei of the cells are labelled by Hoechst staining (grey). Left panel: merged image. Middle and right panels: single channels merged with Hoechst label. Scale bar: 10 μm. (**d**) Nuclear dUTPase staining could be observed in DCX−/GFAP− cells (putative transit amplifying cells, white asterisk). (**e**) Nuclear and cytoplasmal dUTPase staining could be detected in DCX+/GFAP− cells (neuroblasts, yellow asterisks). Individual images were captured with a Nikon AR1 confocal microscope, and the figure was assembled using Adobe Illustrator (Adobe).

**Figure 6:**
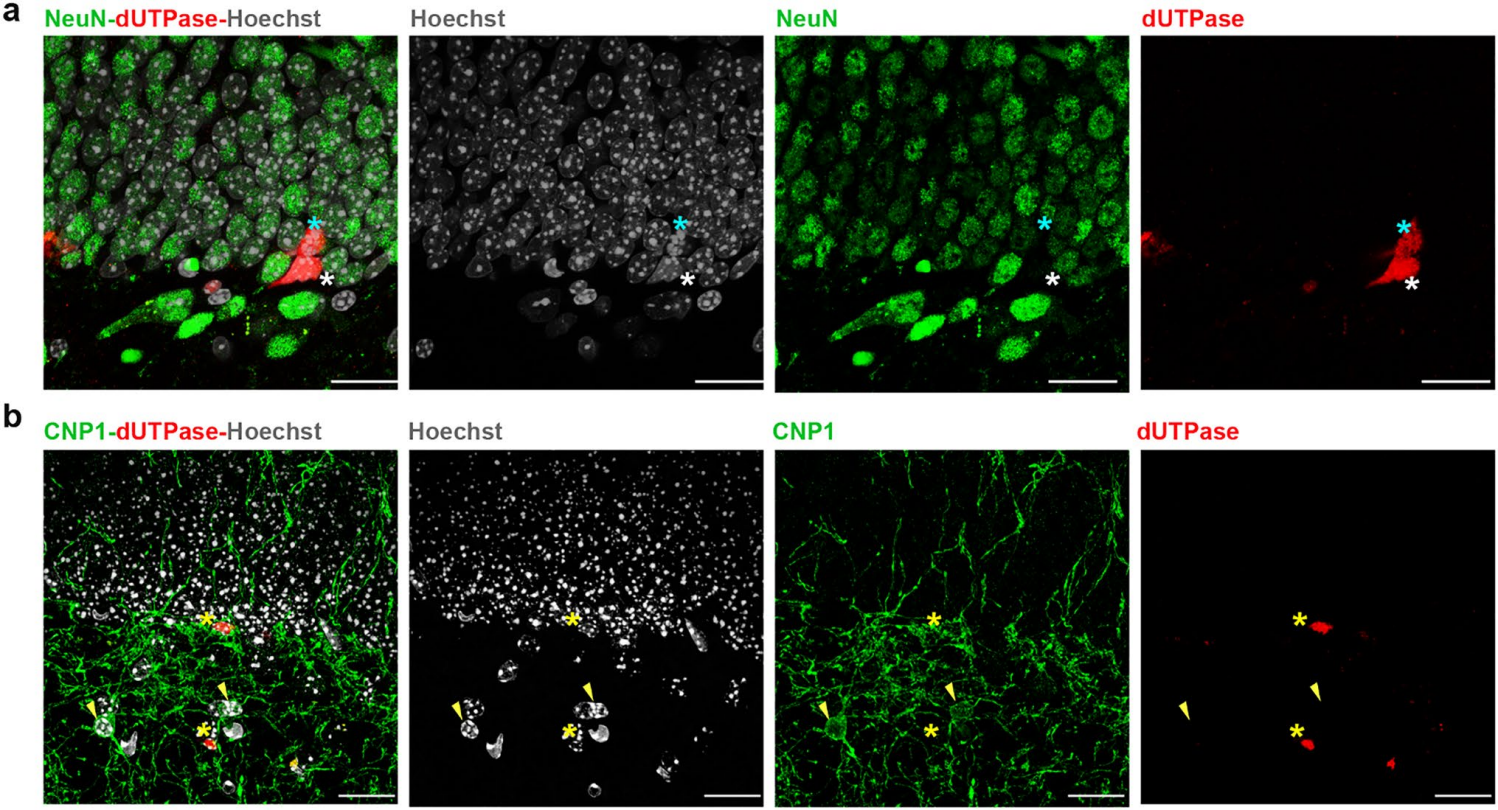
Mature neurons and oligodendrocytes in the dentate gyrus do not express the dUTPase protein. (**a**) NeuN immunopositive (green) mature neurons in the dentate gyrus do not express the dUTPase protein. dUTPase immunopositive (red) cells in the subgranular zone (SGZ) are labelled by asterisks. A dUTPase positive, mitotic cell with condensed chromatin structure is labelled by blue asterisk. Cell nuclei are labelled with Hoechst staining (grey). Scale bar: 20 μm. (**b**) CNP1 immunopositive (green) oligodendrocytes (yellow arrowheads) in the dentate gyrus do not express the dUTPase protein. dUTPase positive cells (red) in the SGZ are labelled by yellow asterisks. Cell nuclei are labelled with Hoechst staining (grey). Scale bar: 20 μm. Individual images were captured with a Nikon AR1 confocal microscope, and the figure was assembled using Adobe Illustrator (Adobe).

We investigated the cellular composition of the germinative zones in the adult mouse brain, the subventricular zone (Fig. 4) and the subgranular zone (Fig. 5). To discriminate between the different cell types located in the germinative zones (Figs. 4b, 5b), we applied immunostaining for markers selective for distinct progenitor types together with the dUTPase staining. We used the glial fibrillary acidic protein (GFAP), which is expressed in relatively quiescent neural stem cells, (type B1 and type 1 cells for the SVZ and SGZ, respectively) and doublecortin (DCX), which is expressed in the post-mitotic neuroblasts (type A and type 3 cells for the SVZ and SGZ, respectively)^88–90^. We also aimed to investigate oligodendrocyte progenitors (OPCs) which originate from restricted periventricular germinal regions at embryonic and postnatal ages and migrate to the gray and white brain matter where they can differentiate into oligodendrocytes until adulthood^91^. We used Neural/glial antigen 2 (NG2) to identify OPCs and CNP1 to identify mature oligodendrocytes. Neuronal nuclei protein (NeuN) was used as a post-mitotic neuronal marker^92^. We found that immunoreactivity of dUTPase could not be detected in GFAP+ cells (Figs. 4d–f, 5d). In a subpopulation of the DCX−/GFAP− cells dUTPase staining could be observed in the nuclear compartment (Figs. 4d–f, 5d). As neither NeuN positive mature neurons (Fig. 6a) nor CNP1 containing oligodendrocytes (Fig. 6b) neighbouring the germinative zones were positively stained for the dUTPase protein, and we could not detect NG2 oligodendrocyte progenitors in the SVZ or SGZ, we assume that the DCX−/GFAP− population in the neurogenic niches represents the intensively proliferating intermediate progenitor cells derived from the neural stem cell pool (type C and type 2 cells for the SVZ and SGZ, respectively). Although, we cannot completely exclude the presence of some DCX−/GFAP− microglial cells in this region^93^. Furthermore, dUTPase protein could be detected in the mitotic DCX−/GFAP− cells characterized by condensed chromatin (Fig. 4e,f). DCX+ postmitotic neuroblasts contained dUTPase both in their nuclear and cytoplasmal compartments (Figs. 4e, 5e). These data suggest that while the relatively quiescent neural stem cell population lacks dUTPase protein, the enzyme starts to appear in the intensely proliferating intermediate progenitor cells, presumably around the start of the mitotic activity and is present during the mitosis. However, the presence of dUTPase in the proliferating stem cells cannot be excluded based on our data as we could not find GFAP+ mitotic cells in our samples presumably due to their low frequency. Postmitotic neuroblasts keep on expressing the dUTPase protein, which partially dislocates to their cytoplasm. The dUTPase protein expression is sustained in the migrating neuroblasts throughout the RMS. Supporting our observation, Cebrian-Silla et al. determined the expression of different genes by single-cell RNA sequencing in the SVZ of the adult mouse brain, and based on their data, dUTPase was expressed in dividing cells, including type A, B and C cells^94^. The expression of dUTPase was elevated in type A cells expressing DCX, and also in dividing type B and C cells. Taken together, our results underline the presence of dUTPase in dividing cells in adult neurogenesis.

**Figure 7:**
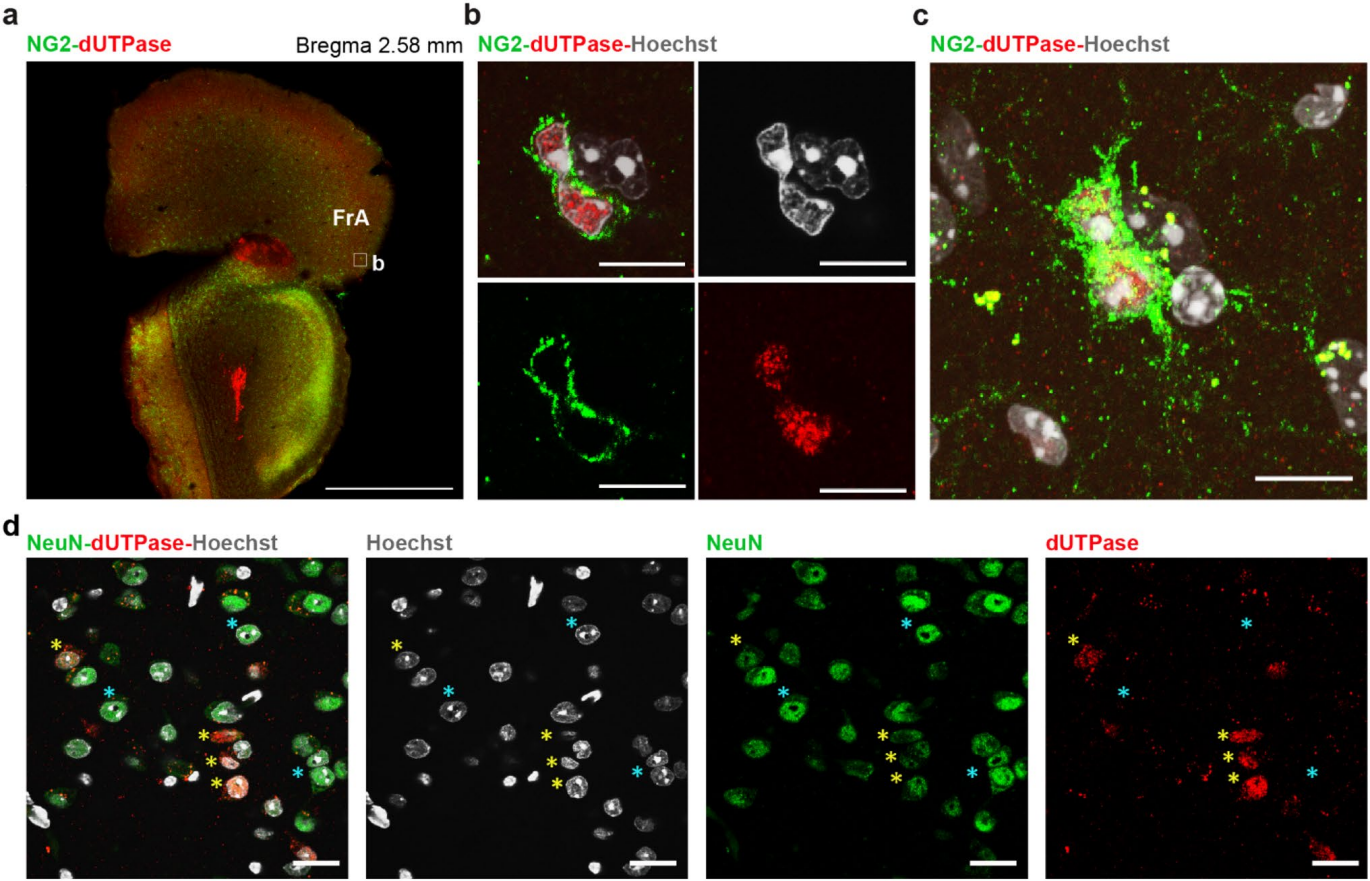
Expression of dUTPase in subcortical structures. (**a**) Low resolution confocal image of a coronal brain section containing the frontal association cortex (FrA). Oligodendrocyte progenitors are stained by NG2 (green), dUTPase protein is stained in red. Region of interest (ROI) of (**b,c**) is indicated by white square. Scale bar: 500 μm. (**b**) Single optical section of a high resolution confocal image. dUTPase protein (red) is present in the nucleus (Hoechst staining, grey) of a NG2 immunpositive (green) cell. Scale bar: 20 μm. (**c**) Same as on (**b**) higher left panel. Merge of multiple optical sections, where the characteristic bipolar morphology and the multiple processes of the oligodendrocyte progenitor can be observed. Scale bar: 20 μm. (**d**) Neurons labelled by NeuN (green) in the central amygdala. Fraction of neurons (yellow arrowheads) contained dUTPase protein (red) in their nuclei (Hoechst staining, grey). Neurons with no detectable dUTPase content are labelled by blue asterisks. Scale bar: 20 μm. Individual images were captured with a Nikon AR1 confocal microscope, and the figure was assembled using Adobe Illustrator (Adobe).

Faint nuclear dUTPase staining could be also found throughout the brain in a small portion of NG2-positive oligodendrocyte progenitors (Fig. 7a–c) as well as in a fraction of mature neurons of certain subcortical brain areas including central amygdala (Fig. 7d), habenula, intralaminar and midline thalamic nuclei, dorsal raphe and locus coeruleus. We could not detect dUTPase staining in astrocytes (GFAP+) or oligodendrocytes (CNP1+).

According to transcriptome databases dUTPase mRNA expression is elevated in neural progenitor cells, oligodendrocyte precursor cells and neurons^95–97^. Our findings underline these data as dUTPase protein expression was observed in intensely proliferating intermediate progenitor cells, migrating neuroblasts, oligodendrocyte progenitors and in a fraction of mature neurons.

The maintenance of genomic integrity is important in the intensely proliferating NPCs and postmitotic neurons, as neuronal function is extensively affected by DNA damage. It is concluded that the activity of BER in the brain is the highest during neurogenesis^98^. It was also demonstrated that repair of DNA damage associated with uracil misincorporation is critical for neuronal survival^99^. The key role of dUTPase is to maintain cellular dUTP:dTTP ratio to prevent uracil misincorporation and subsequent uracil excision by BER. As the methylation of dUMP to dTMP requires folic acid, under folate depletion the intracellular dUTP level increases. Interestingly, it was demonstrated that folate deficiency strongly repressed neurogenesis^100^ and inhibited proliferation of adult hippocampal progenitors^101^. Accordingly, the observed expression of dUTPase during neurogenesis suggests that the elimination of dUTP and the biosynthesis of dTTP is of utmost important in neuronal function.

## Conclusion

Mouse is a widely used model organism to study mammalian development. In this work, we investigated the isoform-specific mRNA expression of dUTPase with RT-qPCR in various organs of mice during development. We analysed brain, gonad and liver derived from 14.5-day embryos, moreover, heart, kidney, lung, spleen and thymus were also investigated in 2-week-old, 4-week-old, 10-week-old and 13-month-old mice. This experimental set-up represents the three germ layers: brain (ectoderm); heart, kidney, ovary and testis (mesoderm); thymus, lung and liver (endoderm). The nuclear isoform of dUTPase is more abundant than the mitochondrial isoform based on their mRNA expression. In addition, the changes observed during development and among the organs show larger differences in case of the nuclear isoform.

We found that expression of nDut in the lymphoid organs, i.e., the thymus and spleen, shows uniformly high level throughout development. It may be explained by the fact that maturation of thymocytes occurs in the thymus and immature thymocytes reside there^54^. Similarly, in the spleen early maturation and differentiation process of B cells occurs^55^. Regarding the reproductive organs, we found elevated mRNA expression of nDut, as folliculogenesis and spermatogenesis occur in the gonads. Nevertheless, differences can be observed between the sexes at the 4-week-old stage and after, notably the expression of nDut in the adult testis is threefold higher than in the adult ovary. The reason for this may be that although during spermatogenesis continuous renewal of spermatocytes occurs in the testis^67,68^, no mitotically active female germline progenitors exist in postnatal mouse ovaries^65,66^. During embryonic liver development hepatogenesis occurs^57,58^, accordingly, the expression of nDut is 20-fold higher in the E14.5 samples than in the 4- and 10-week-old groups. The latter groups represent the ones with the lowest expression among the organs investigated. Data obtained from single-cell RNA sequencing analysis of several mouse organs reinforce our results as elevated *Dut* expression was detected in immature T cells in the thymus, in B cells in the spleen, in spermatocytes in the testis, in cumulus cells in the ovum, in hepatocytes in the liver and in neurons in the brain^95,97^. These observations underline that dUTPase has an important function in cell proliferation and presumably in cell differentiation, as well. In some 13-month-old tissues we observed a slight increase in the expression of *Dut* isoforms, notably in the brain and gonad samples. Various studies have reported an age-related decline in DNA repair processes, importantly in the BER pathway in mouse brain and germ cells^29,72,102^. During aging the activity and expression of DNA glycosylases, particularly uracil-DNA-glycosylases was shown to decrease. Accordingly, we propose that dUTPase can act as a compensatory enzyme by preventing dUTP nucleotide misincorporation, hence attempt to maintain genomic integrity during aging.

Moreover, we found that the expression of nDut in the adult mouse brain is relatively high. This finding is rather surprising as dUTPase has been shown to play an important role in proliferating cells and the mass production of neural cells is completed by adulthood. However, the importance of appropriate dTTP biosynthesis was described in neuronal development, since in human and in zebrafish erroneous DTYMK led to severe postnatal neurodegenerative disease^16,17^. Therefore, we investigated the dUTPase protein expression in 3-month-old adult mouse brain in further details using immunostaining. Our results showed that dUTPase expression is mainly observed in the progenitor cells in the germinative zones, i.e., in the subventricular and the subgranular zone. We showed that dUTPase is expressed in the intensely proliferating intermediate progenitor cells, presumably from the start of the mitotic activity, and is present during mitosis. Postmitotic neuroblasts also keep on expressing the enzyme, which partially dislocates to their cytoplasm. Importantly, we detected dUTPase expression in the migrating neuroblasts throughout the RMS. The elevated dUTPase protein expression in these regions may account for the measured high mRNA level, as estimates suggest that several thousand neural cells are generated in the different compartments of the young adult mouse brain^86^. In addition, we found scattered dUTPase labelling in a small portion of the oligodendrocyte progenitors throughout the brain. Faint dUTPase staining could be observed in a subset of mature neurons in defined subcortical structures. As it was shown that DNA damage and repair events play an important role in the physiological functions of neurons^103–105^, the presence of the dUTPase protein may reflect the level of metabolic or neuronal activity in certain brain regions.

Developmental role of dUTPase was already suggested in *Drosophila melanogaster* where it was shown that the expression of the enzyme is under strict developmental control both in the embryo and in the larval stages^106,107^. From another, but related point of view, recent data argued strongly for an important role of thymidylate biosynthesis in the embryonic development of zebrafish with a special role in brain development^16,17^. Our present data expand these observations to the mouse model, as well. Next to our extensive isoforms-specific gene expression analysis in the different mouse organs during development, we showed that dUTPase protein expression in the adult mouse brain is mainly observed in the progenitor cells in the germinative zones and the rostral migratory stream. Our results suggest that dUTPase expression is critical during cell differentiation and development.

## Materials and methods

### Animals

Mice (FVB/N background) were produced and maintained in the Animal Care Facility of the Department of Animal Biotechnology, Institute of Genetics and Biotechnology, Hungarian University of Agriculture and Life Sciences in Gödöllő. Animals were housed in groups of 2–5 with free access to food and water and were kept under standard light–dark cycle (06.00–18.00 h) at 22 °C. This study was carried out in strict accordance with the recommendations and rules of the Hungarian Code of Practice for the Care and Use of Animals for Scientific Purposes. For the experiments, 56 FVB/N mice were used, including 7 14.5-day embryos and 7 animals for each developmental stage and sex, excluding the 13-month-old stage, where only male animals were used. In case of the embryos and the 2-week-old young animals weight measurement was inaccessible. In case of the other age classes, 7 female mice of 4 weeks of age with an average weight of 16.4 ± 0.4 g and 7 female mice of 10 weeks of age with an average weight of 20.4 ± 0.5 g were used. Regarding the male mice, 7 animals of 4 weeks of age with an average weight of 19.1 ± 0.3 g, 7 animals of 10 weeks of age with an average weight of 26.9 ± 1.0 g and 7 animals of 13 months of age with an average weight of 30.7 ± 1.4 g were used. Uncertainty is indicated with standard deviation. Cervical dislocation was the method of choice for euthanasia. All efforts were made to minimize suffering. Brain, gonad and liver samples were collected from 14.5-day embryos. Brain, gonad (ovary or testis), liver, kidney, lung, spleen and thymus samples were collected from 2-week-old, 4-week-old, 10-week-old mice from both sexes, while the same organs were collected from 13-month-old male mice. Mouse organs were prepared by macrodissection and were quickly frozen in liquid nitrogen and stored at − 70 °C until processing. After sample collection RNA isolation was carried out within a month. We performed all experiments on animal materials postmortem; however, we report our results in accordance with the relevant points of the ARRIVE guidelines. For the immunohistology experiments, mice (C57Bl/6J background) were produced and maintained in the Institute of Experimental Medicine, Budapest, in accordance with the regulations of the European Community’s Council Directive of November 24, 1986 (86/609/EEC). We performed all experiments on animal materials postmortem. Experiments were approved by Animal Welfare Committee of the Institute of Experimental Medicine and the National Animal Research Authorities of Hungary (PE/EA/877-7/2020).

### RT-qPCR method

The RT-qPCR measurements were carried out using a previously described and optimized RT-qPCR method^5^. Briefly, RNA isolation from various organs was performed using RNeasy Plus Mini Kit (Qiagen) and RNase-Free DNase Set (Qiagen) according to the manufacturer’s instructions. The concentration and purity of the RNA samples were determined with NanoDrop ND-1000. The 260/280 absorbance ratio values for the samples of the 14.5-day embryos and of the 2-week-old, 4-week-old, 10-week-old and 13-month-old mice are summarized in Supplementary Table S1. The data for the 10-week-old samples were previously described^5^. The integrity of the RNA samples and possible contamination with genomic DNA were assessed with agarose gel electrophoresis (Supplementary Fig. S2) and with direct qPCR analysis of the control RNA samples without reverse transcription (Supplementary Fig. S1). RNA samples were subjected to cDNA synthesis using the High-Capacity cDNA Reverse Transcription Kit (Applied Biosystems) according to the manufacturer’s recommendations. For the no reverse transcriptase control (NRT) samples no reverse transcriptase enzyme were added. For the qPCR amplification MyTaq HS Mix (Bioline), Evagreen dye (Biotium) and nuclease-free water (Ambion) were used. The primers (Sigma) used in this study were described previously^5^. GAPDH-Fw and GAPDH-Rev primers were used in a final concentration of 315 nM, PPIA-Fw and PPIA-Rev primers were used in a final concentration of 425 nM and 500 nM, respectively. 1200 nM final concentration was used for nDut-Fw primer, 375 nM final concentration was used for mDut-Fw primer and 1000 nM final concentration was used for Rev1 primer. For the qPCR measurements Clear Hard-Shell 96-Well PCR Plates (Bio-Rad) and Microseal ‘B’ PCR Plate Sealing Film (Bio-Rad) were used. cDNA samples derived from 200 ng of RNA isolated from 8 different organs (brain, heart, kidney, liver, lung, gonad/ovary/testis, spleen and thymus) of male and female mice were subjected to qPCR in a final dilution of 0.31 µl/10 µl reaction. A gene maximizing plate design was used with manual reaction setup. At least three biological replicate cDNA samples were measured for each biological group. Three technical replicates were used for each reaction. Four replicates of no template controls (NTC) for each target were also included in each plate. Three technical replicates of no reverse transcriptase controls (NRT) were applied for each target in the case of approximately 55% of the samples. The PCR reactions were performed as following: activation and denaturation at 95 °C for 5 min, followed by 50 cycles of denaturation at 95 °C for 30 s, annealing and extension at 66 °C for 30 s, then melting curve analysis from 60 to 95 °C at a rate of 0.5 °C/5 s using CFX96 real-time PCR detection system (Bio-Rad).

### Immunostaining

3-month-old mice were perfused with 4% paraformaldehyde (TAAB Laboratory, #P001) and 0.1% glutaraldehyde (Electron Microscopy Sciences, #16210) in 0.1 M PB. Coronal section (50 μm thick) were cut with vibratome. To permeabilize the membranes sections were incubated in sucrose (30%) overnight, followed by freeze-thawing over liquid nitrogen. For initial screening in the adult brain tissue (data not shown), dUTPase was visualized with a rat anti-dUTPase antibody (1:5000, Sigma-Aldrich, #SAB4200044) followed by biotinylated rabbit anti-rat (1:300, Vector Laboratories, #BA-4001-0.5) and avidin biotinylated horseradish peroxidase complex (ABC, 1:300, Vector Laboratories, #PK-4000). Nickel-intensified 3,3′-diaminobenzidine (DABNi, bluish-black reaction product, DAB: Sigma-Aldrich, #D5637) was used as a chromogen. For confocal analysis, dUTPase was visualized with a rat anti-dUTPase antibody (1:3000, Sigma-Aldrich, #SAB4200044) followed by anti-rat-Cy3 antibody (1:500, Jackson, #AB_2340667). DCX was stained by guinea pig anti-DCX antibody (1:1000, Millipore, AB2253) followed by a goat anti-guinea pig-Alexa 647 (1:500, Molecular Probes, #A-21450) secondary antibody. GFAP was stained by a mouse anti-GFAP antibody (1:1000, Sigma-Aldrich, G3893-0.2 ML) followed by a goat anti-mouse-Alexa 488 (1:500, Molecular Probes, #A28175) secondary antibody. NG2 was stained by a rabbit anti-NG2 antibody (1:100, Sigma-Aldrich, #ZRB5320) followed by a donkey anti-rabbit-Alexa 488 (1:500, Invitrogen, #A-21206). CNP1 was stained with rabbit anti-CNP1 antibody, courtesy of Ádám Dénes (1:1000, Synaptic Systems) followed by donkey anti-rabbit-Alexa 488 (1:500, Invitrogen, #A-21206). NeuN was stained by mouse anti-NeuN antibody (1:3000, Sigma-Aldrich, #MAB377) followed by donkey anti-mouse-Alexa 488 (1:500, #AB2341099). Nuclei were stained via Hoechst dye (Sigma-Aldrich, #14533). Results were obtained with a Nikon AR1 confocal microscope. At some Figures colors of the fluorescent channels were subsequently modified digitally.

### Data analysis

Cq values were identified as outlier and disposed of if the relative fluorescence unit (RFU) value was lower by 10% than the average final RFU value for the corresponding target. In addition, if the melting curve analysis showed aspecific product formation, the data point was also identified as an outlier. Approximately 5% of the data were excluded from the analysis based on these criteria. Moreover, the differences in the Cq values between each sample and their respective NTC/NRT controls were more than 5 cycles, however, in most cases, it was more than 8 cycles, thus, all results were accepted in this regard.

To determine the relative normalized expression (RNE), we used two reference genes, *Gapdh* and *Ppia*, and their expression was defined as ideal according to the CFX Maestro 2.0 software (Bio-Rad). For the determination of the RNE we analysed the biological groups defined by the age and sex for each organ and compared the expression values to the 10-week-old male samples in every case. The relative normalised expression values measured during development were corrected with a factor, which is the relative expression value of the 10-week-old organs described previously^5^. The relative normalized expression data were transferred from CFX Maestro 2.0 to Microsoft Excel (Microsoft Office). The expression data, the standard deviation (SD), the mean Cq values of the technical replicates, the factor and the corrected expression values of all biological replicate samples for each isoform are summarized in Supplementary Table S1. The expression and the corrected expression data with the corresponding standard deviation error values of the biological groups are also summarized in Supplementary Table S1. Regarding the expression of the embryonic gonad, we calculated the corrected expression values for male and female reproductive organs separately in case of each sample, then we composed the corrected expression of the E14.5 gonad samples as the average of the expression calculated for the different sexes.

The statistical analysis was performed using XLSTAT (Lumivero). As the variance of the biological replicate samples for the organs measured during development is not equal, we performed non-parametric Kruskal–Wallis analysis followed by Conover-Iman pairwise comparisons with Bonferroni correction using an overall 5% significance level. The Bonferroni corrected significance level in case of the brain, gonad and liver was 0.0018 as E14.5 samples were also analysed, while in case of the heart, kidney, lung, spleen and thymus it was 0.0024.

### Softwares used

Amplification curves and melting curves were illustrated by CFX Maestro 2.0 software (Bio-Rad). Cq determination mode was set to single threshold with a fixed threshold set to 500 RFU for each plate. Gel images were captured with Image Lab 4.1 software (Bio-Rad). Bar graphs were created with OriginPro 2018 (OriginLab Corp.). CorelDRAW Graphics Suite 2021 (Corel Corporation) and Adobe Illustrator (Adobe) were used for creating figures from individual graphs. Images of organs were downloaded from Adobe Stock. The statistical analysis was performed using XLSTAT (Lumivero) in Microsoft Excel (Microsoft Office).

### Supplementary Information

Supplementary Table S1.

Supplementary Information.

## Data Availability

No datasets were generated or analysed during the current study. Raw data is available upon request (e-mail: vertessy.beata@ttk.hu, nagy.nikolett@ttk.hu).
