# Supplementary material for: Characterization of dUTPase expression in mouse postnatal development and adult neurogenesis
Source: Sci Rep. 2024 Jun 7;14:13139. doi: 10.1038/s41598-024-63405-0 (PMC11161619; doi:10.1038/s41598-024-63405-0)
Supplement: Supplementary file 2 — Supplementary Information. [file 41598_2024_63405_MOESM2_ESM.docx]

**Supplementary Information**

**Characterization of dUTPase expression in mouse postnatal development and adult neurogenesis**

Nikolett Nagy^1,2*#^, Nóra Hádinger^3#^, Otília Tóth^2,4^, Gergely Attila Rácz^2,4^, Tímea Pintér^5^, Zoltán Gál^5^, Martin Urbán^5^, Elen Gócza^5^, László Hiripi^5,6^, László Acsády^3^, Beáta G. Vértessy^2,4*^

Affiliations

^1^Doctoral School of Biology, Institute of Biology, ELTE Eötvös Loránd University, 1117 Budapest Pázmány Péter sétány 1/C, Budapest, Hungary

^2^Institute of Molecular Life Sciences, Research Centre for Natural Sciences, HUN-REN, 1117 Magyar tudósok körútja 2, Budapest, Hungary

^3^Laboratory of Thalamus Research, Institute of Experimental Medicine, HUN-REN, 1083 Szigony utca 43, Budapest, Hungary

^4^Department of Applied Biotechnology and Food Sciences, Faculty of Chemical Technology and Biotechnology, BME Budapest University of Technology and Economics, 1111 Műegyetem rkp. 3, Budapest, Hungary

^5^Department of Animal Biotechnology, Institute of Genetics and Biotechnology, Hungarian University of Agriculture and Life Sciences, 2100 Szent-Györgyi Albert utca 4, Gödöllő, Hungary

^6^Laboratory Animal Science Coordination Center, Semmelweis University, 1089 Nagyvárad tér 4, Budapest, Hungary

*Corresponding authors

#These authors contributed equally to this work

Correspondence and requests for materials should be addressed to Beáta G. Vértessy (e-mail: [vertessy.beata@ttk.hu](mailto:vertessy.beata@ttk.hu)). Correspondence may also be addressed to Nikolett Nagy (e-mail: [nagy.nikolett@ttk.hu](mailto:nagy.nikolett@ttk.hu)).

**Supplementary Table S1.** Expression data regarding the biological replicate samples and biological groups. The first worksheet contains data related to the biological replicate samples for each isoform, the 260/280 absorbance ratio of the RNA samples, the expression data with the standard deviation (SD), the mean Cq values of the technical replicates, the factor, and the corrected expression values. Regarding the expression of the embryonic gonad, for each sample we calculated the corrected expression for male and female reproductive organs separately, then we composed the corrected expression of the E14.5 gonad as the average of the expression calculated for the different sexes. The second worksheet contains data related to the biological groups, the mean expression, and the corrected mean expression data with the corresponding standard deviation error values. The blue background represents data related to the mDut, while the red background represents data related to the nDut.


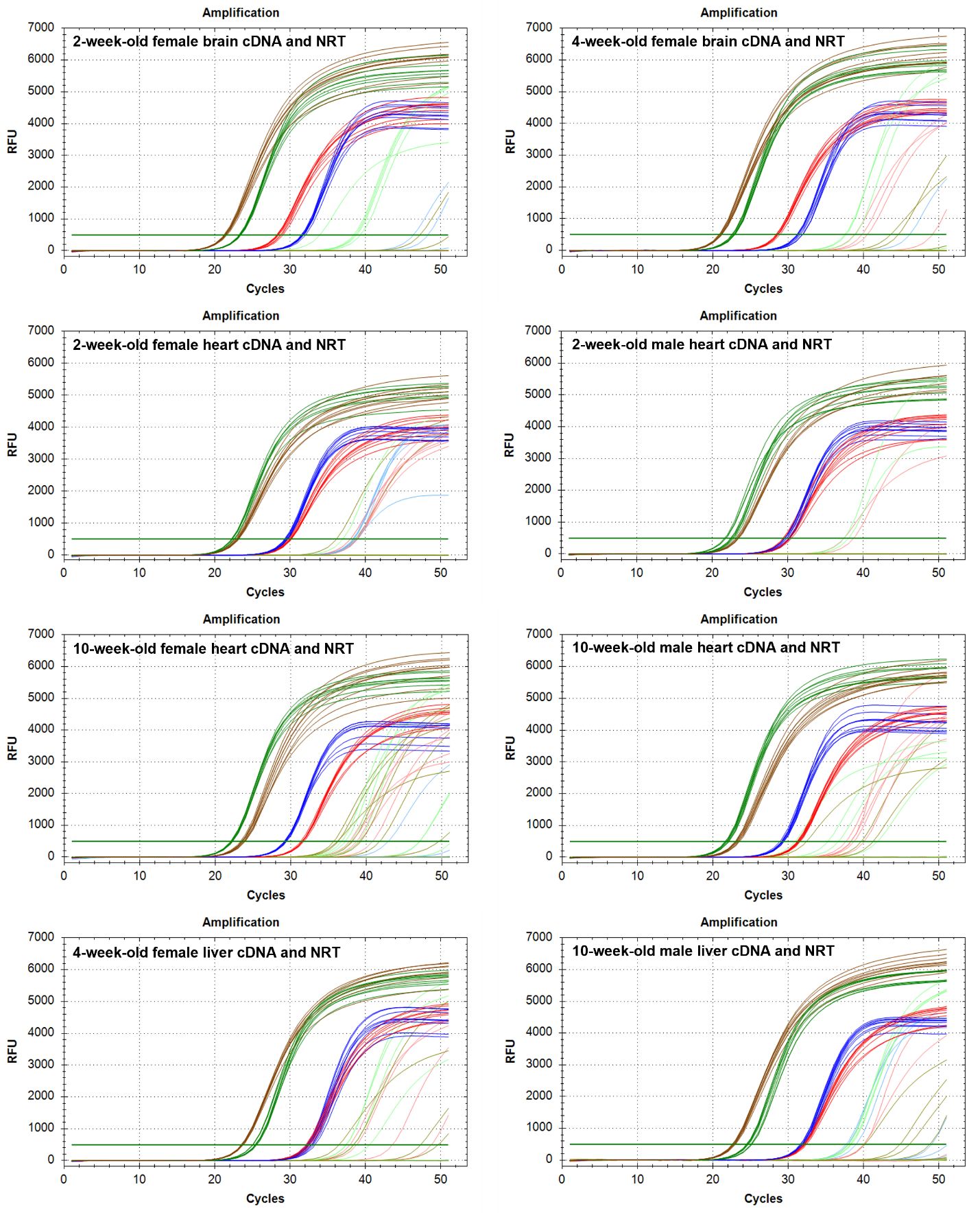


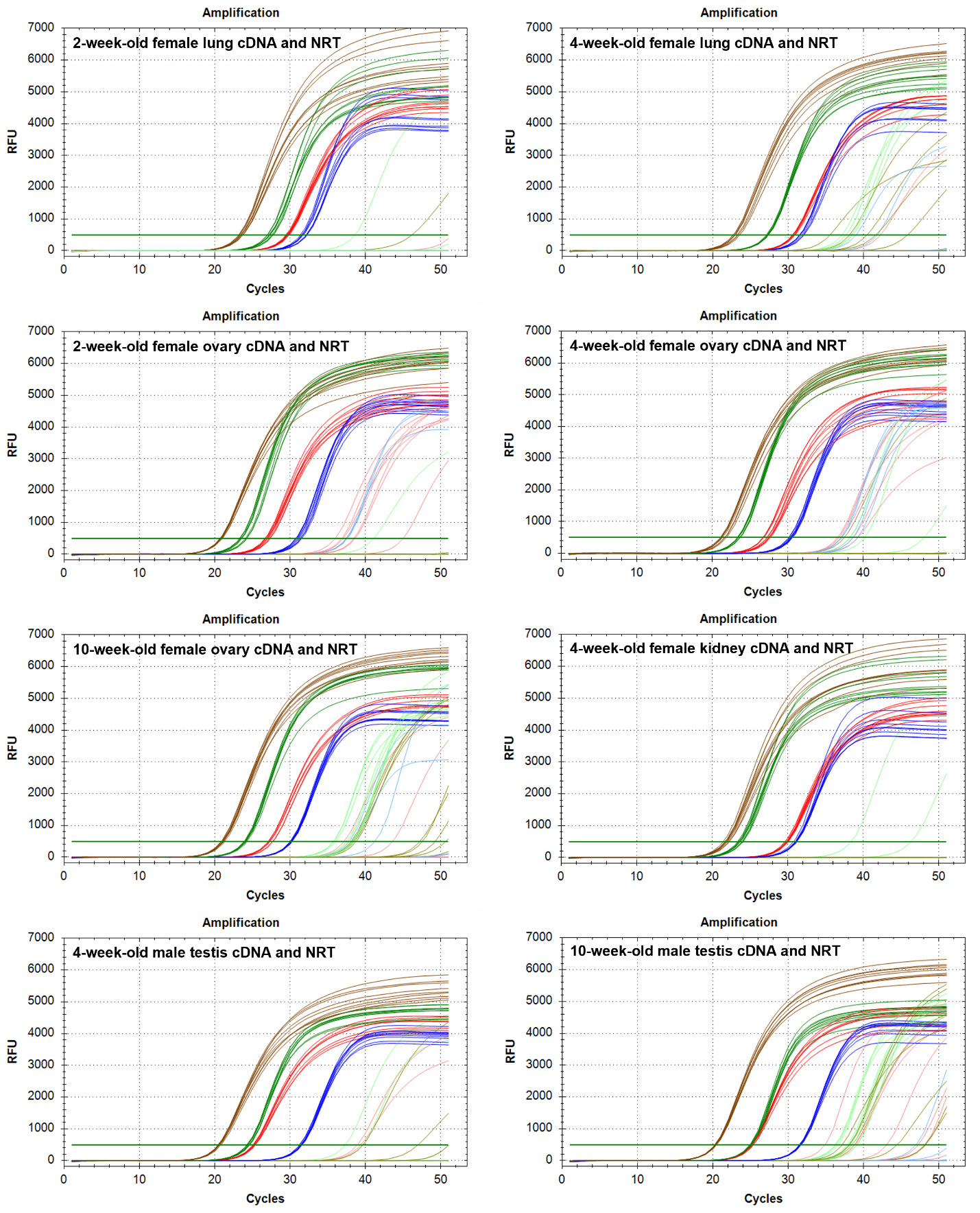


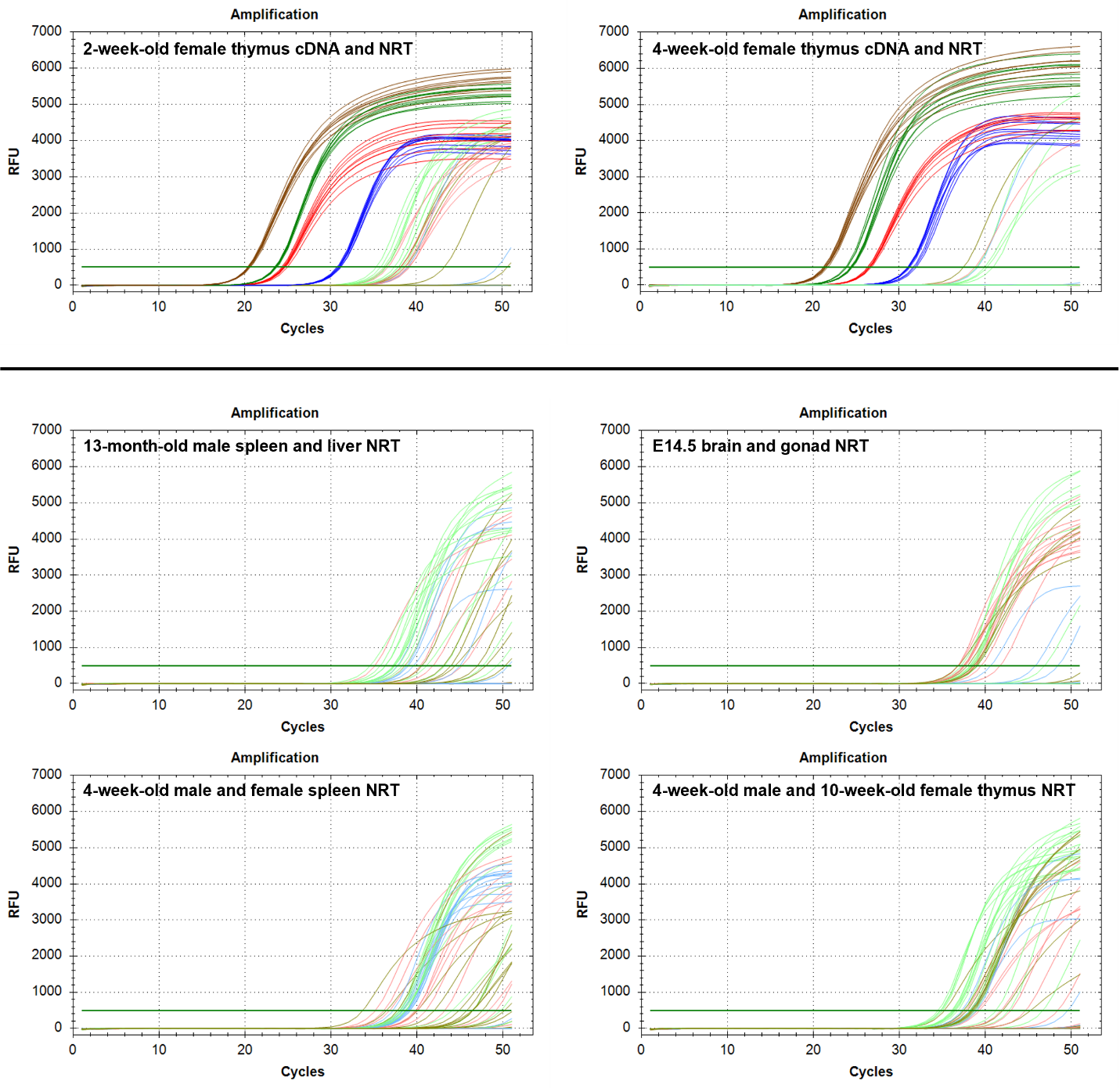


**Supplementary Figure S1.** Representative amplification curves of cDNA and no reverse transcriptase control (NRT) samples. Red indicates nDut, dark blue indicates mDut, dark green indicates Gapdh, brown indicates Ppia expression. Lighter colors indicate NRT control amplification, pink corresponds to nDut, light blue corresponds to mDut, light green corresponds to Gapdh, and olive corresponds to Ppia NRT amplification. RFU: relative fluorescence unit. For each biological group, amplification curves of three biological replicate samples and the corresponding NRT controls measured in three technical replicate reactions are shown for each target. The last four panels show only amplification curves of the NRT controls. Individual graphs were created in CFX Maestro 2.0 software (Bio-Rad), and the figure was assembled in PowerPoint 365 (Microsoft).


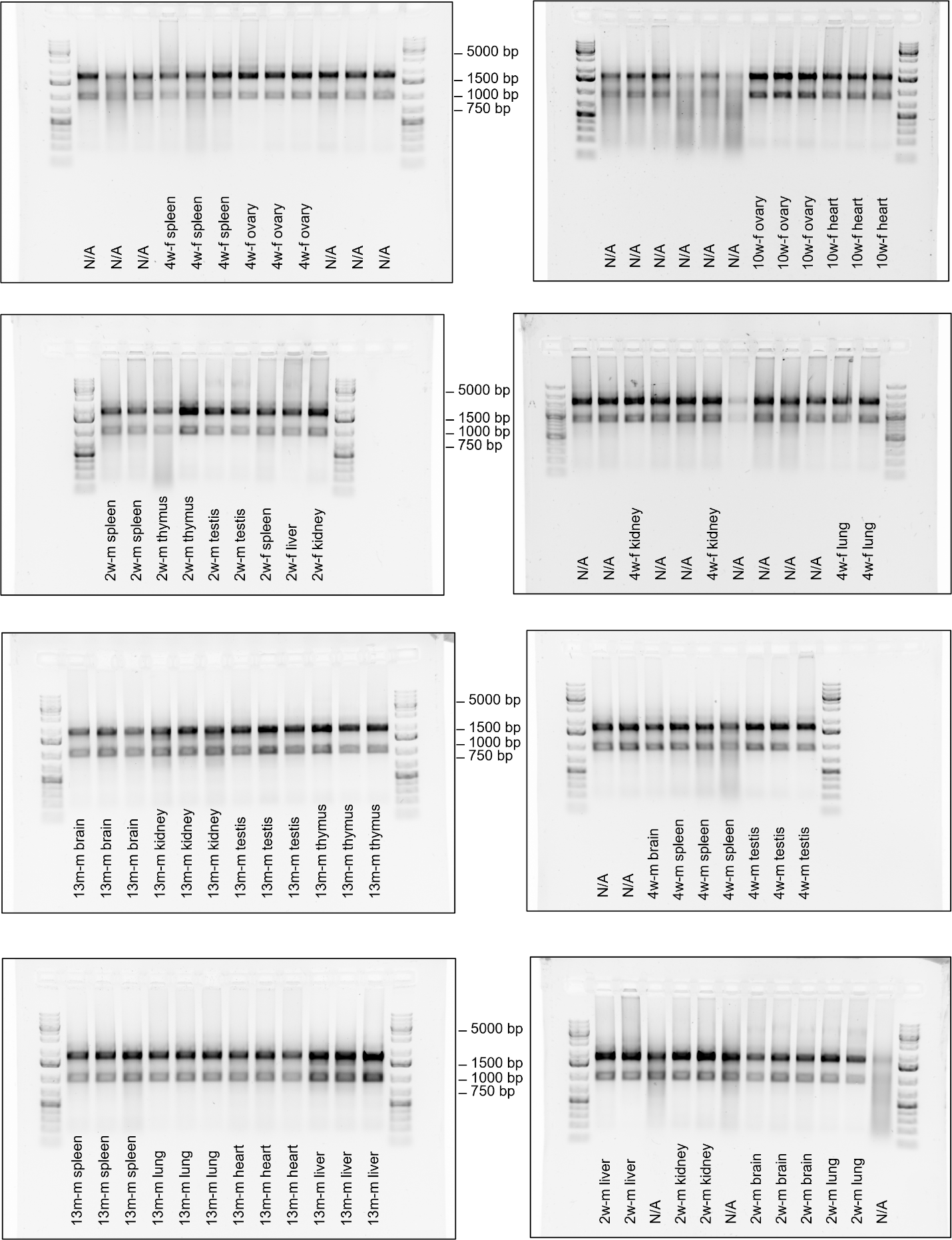


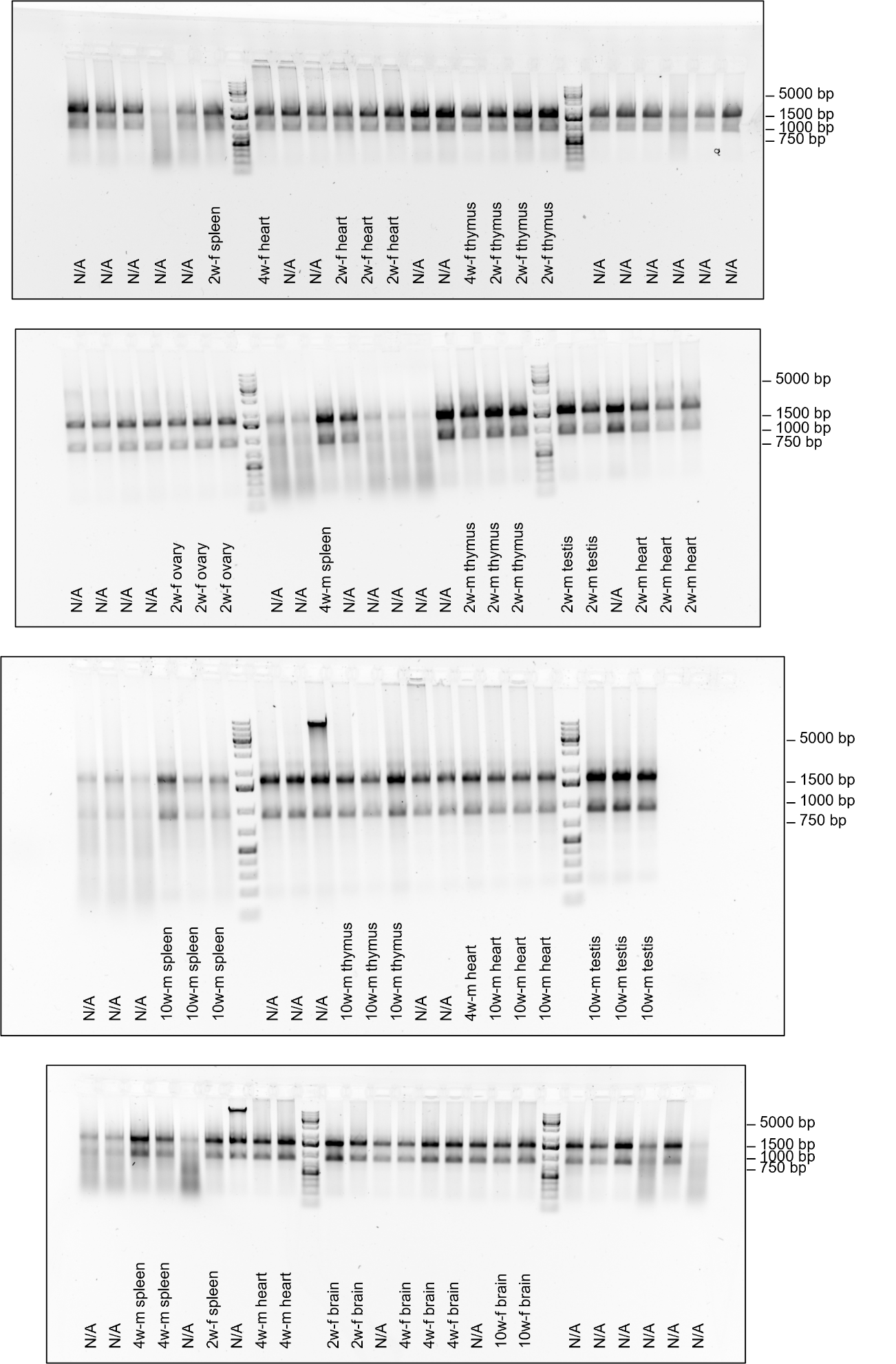

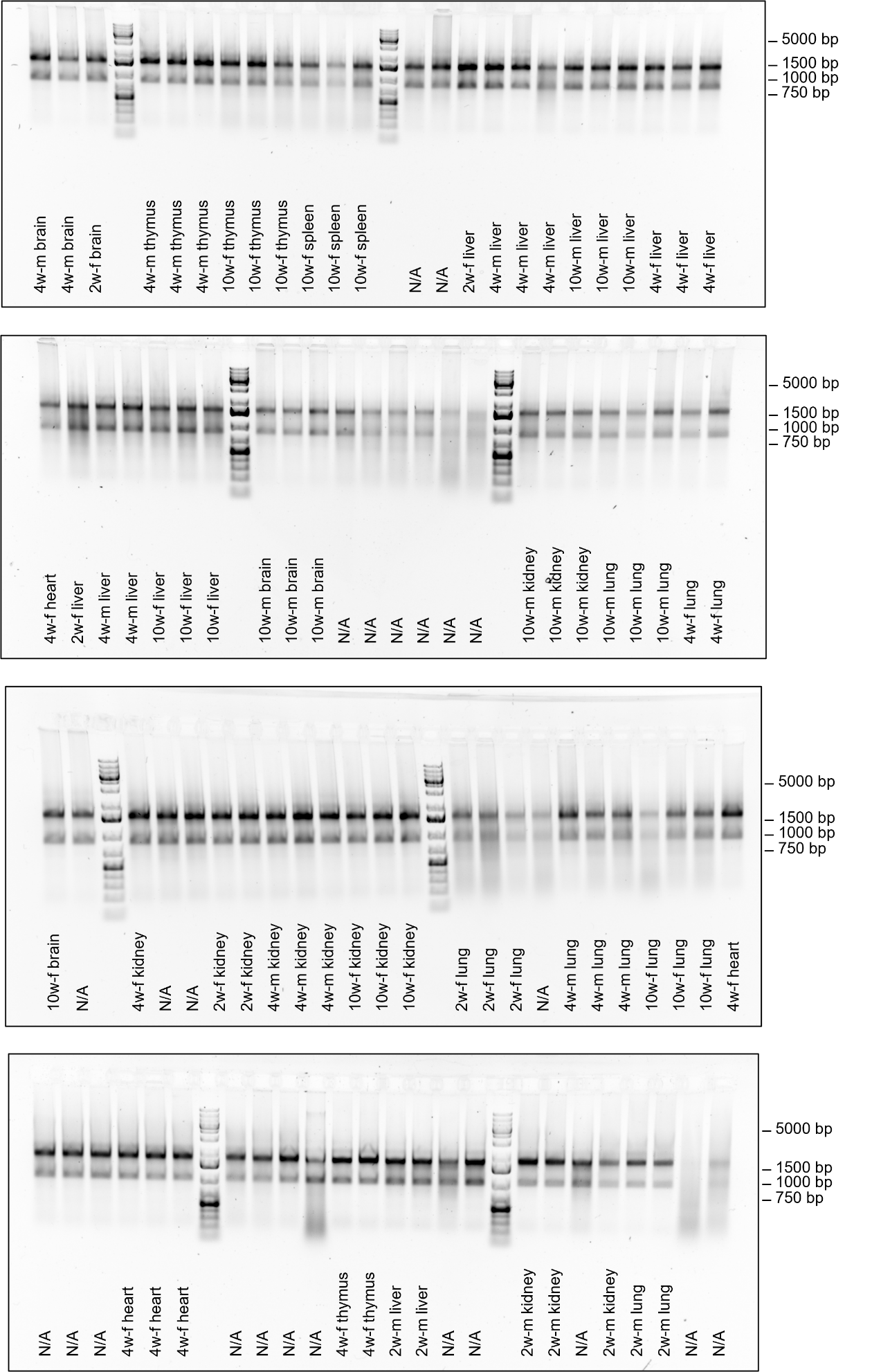

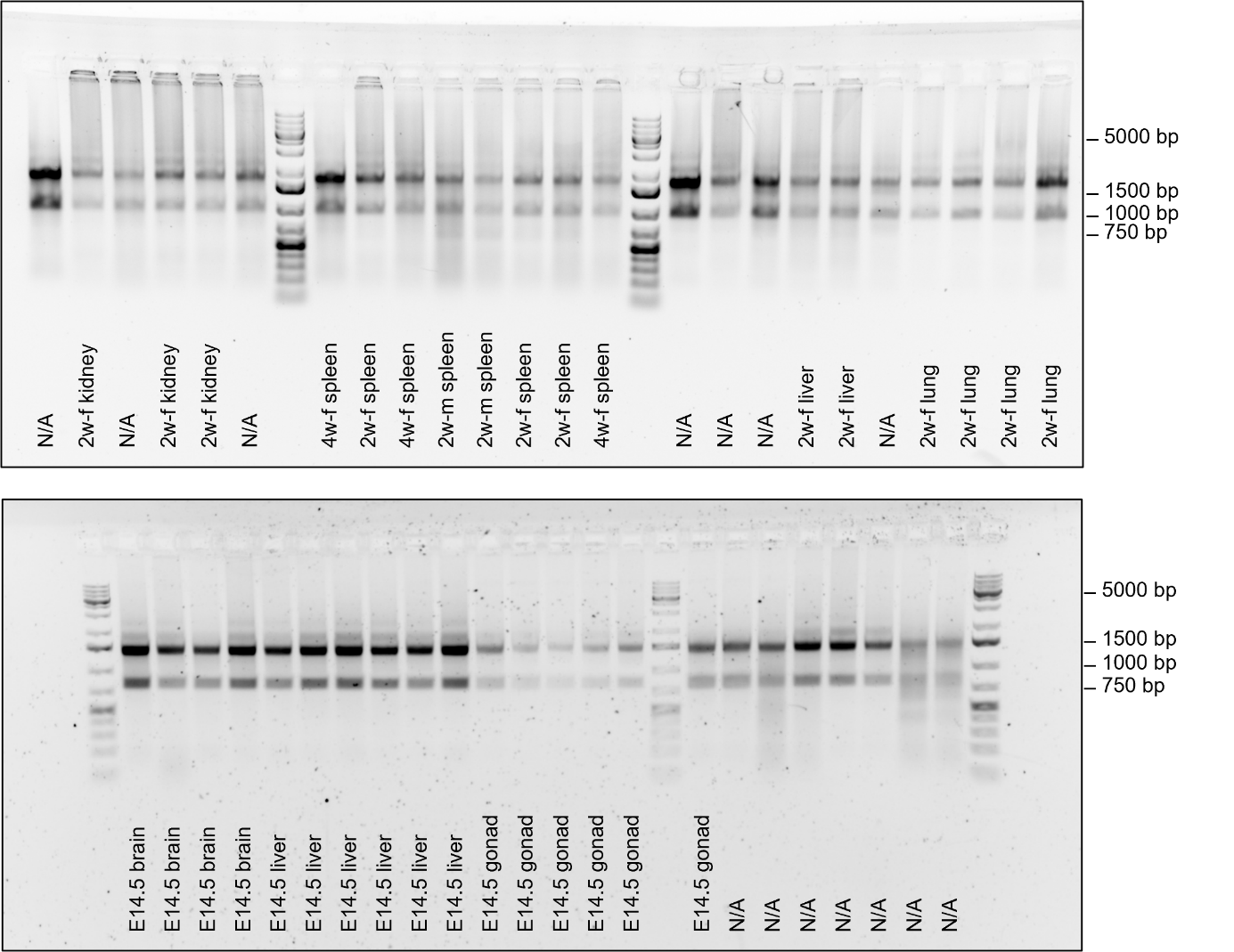


**Supplementary Figure S2.** Agarose gel electrophoresis of the RNA samples. N/A: not applicable.
